# MARCH1 negatively regulates TBK1-mTOR signaling pathway by ubiquitinating TBK1

**DOI:** 10.1186/s12885-024-12667-y

**Published:** 2024-07-26

**Authors:** Xiao Li, Kai Cheng, Meng-Di Shang, Yong Yang, Bin Hu, Xi Wang, Xiao-Dan Wei, Yan-Chun Han, Xiao-Gang Zhang, Meng-Hua Dong, Zhen-Lin Yang, Jiu-Qiang Wang

**Affiliations:** 1 The Second Clinical Medical College , Binzhou Medical University, Yantai, Shandong 264003 P.R. China; 2 Peninsular Cancer Research Center, Binzhou Medical University, Yantai, Shandong 264003 P.R. China; 3 The First School of Clinical Medicine, Binzhou Medical University, Binzhou, Shandong 256603 P.R. China; 4 School of Basic Medical, Binzhou Medical University, Yantai, Shandong 264003 P.R. China; 5 School of Rehabilitation Medicine, Binzhou Medical University, Yantai, 264003 China

**Keywords:** MARCH1, STING, TBK1, mTOR, Growth factors

## Abstract

**Background:**

TBK1 positively regulates the growth factor-mediated mTOR signaling pathway by phosphorylating mTOR. However, it remains unclear how the TBK1-mTOR signaling pathway is regulated. Considering that STING not only interacts with TBK1 but also with MARCH1, we speculated that MARCH1 might regulate the mTOR signaling pathway by targeting TBK1. The aim of this study was to determine whether MARCH1 regulates the mTOR signaling pathway by targeting TBK1.

**Methods:**

The co-immunoprecipitation (Co-IP) assay was used to verify the interaction between MARCH1 with STING or TBK1. The ubiquitination of STING or TBK1 was analyzed using denatured co-immunoprecipitation. The level of proteins detected in the co-immunoprecipitation or denatured co-immunoprecipitation samples were determined by Western blotting. Stable knocked-down cells were constructed by infecting lentivirus bearing the related shRNA sequences. Scratch wound healing and clonogenic cell survival assays were used to detect the migration and proliferation of breast cancer cells.

**Results:**

We showed that MARCH1 played an important role in growth factor-induced the TBK1- mTOR signaling pathway. MARCH1 overexpression attenuated the growth factor-induced activation of mTOR signaling pathway, whereas its deficiency resulted in the opposite effect. Mechanistically, MARCH1 interacted with and promoted the K63-linked ubiquitination of TBK1. This ubiquitination of TBK1 then attenuated its interaction with mTOR, thereby inhibiting the growth factor-induced mTOR signaling pathway. Importantly, faster proliferation induced by MARCH1 deficiency was weakened by mTOR, STING, or TBK1 inhibition.

**Conclusion:**

MARCH1 suppressed growth factors mediated the mTOR signaling pathway by targeting the STING-TBK1-mTOR axis.

**Supplementary Information:**

The online version contains supplementary material available at 10.1186/s12885-024-12667-y.

## Background

mTOR, as a serine/threonine protein kinase, belongs to the PI3K-related kinase family [1] and forms two distinct protein complexes, namely mTORC1 and mTORC2. mTORC1 is composed mainly of RAPTOR (regulatory associated protein of mTOR), mLST8 (also known as GβL), PRAS40 (proline-rich AKT substrate 40 kDa), and mTOR [2–5], while mTORC2 contains mainly Rictor, mSin1, mLST8, and mTOR [1, 6–9]. mTORC1 and mTORC2 both respond to stimulation by growth factors. These growth factors activate mTORC1 via the phosphatidylinositol 3-kinase (PI3K)-AKT signaling pathway. After being activated, mTORC1 phosphorylates its downstream substrates, such as p70S6 Kinase 1 (S6K1) and ULK1 [10, 11]. In response to growth factors, mTORC2 enhances its interaction with ribosomes via PI3K signaling [12], and then phosphorylates and activates downstream substrates, such as the AGC kinases Akt (also known as PKB) and SGK1 [13–15].

TBK1 is a serine/threonine kinase that takes part in diverse cellular processes, including innate immunity and cell survival/proliferation [16–18]. In antiviral innate immunity, cGAS (cGAMP synthase)-STING (a stimulator of IFN genes, also known as MITA, MPYS and ERIS)-TBK1 signaling pathway plays an important role against DNA virus infections [19, 20]. Upon detecting cytoplasmic DNA, STING is activated by cyclic-GMP-AMP (cGAMP) produced by cGAS, and translocates from the endoplasmic reticulum to the ER-Golgi intermediate compartment (ERGIC) [20]. STING then recruits and interacts with TBK1 to phosphorylate interferon regulatory factor 3 (IRF3), subsequently promoting the transcription of type-I interferon [19]. STING can also interact with and be phosphorylated at site Tyr245 by the epidermal growth factor receptor (EGFR), subsequently activating TBK1-IRF3-mediated interferon synthesis [21]. The interaction of STING and TBK1 therefore plays an important role in antiviral immunity. In addition to its role in immunity, aberrant TBK1 activation has been implicated in the oncogenesis of several types of cancer, such as breast and non-small cell lung cancer (NSCLC) [18]. TBK1 promotes cancer cell survival and proliferation by phosphorylating mTOR at Ser 2159 [22, 23]. Moreover, TBK1 also interacts with S6K to regulate the mTORC1 signaling pathway [24]. Although the TBK1-mediated mTOR signaling pathway has been investigated in detail, it remains unclear how the pathway is regulated.

Membrane-associated RING-CH-1 (MARCH1) is a member of the MARCH family of membrane-bound E3 ubiquitin ligases, which play an important role in immunity [25]. MARCH1 ubiquitinates CD86 to promote antigen presentation in dendritic cells (DCs) [25]. In B cells, MARCH1 promotes the ubiquitination of major histocompatibility complex class II (MHC-II) proteins [26]. In response to a human cytomegalovirus (HCMV) infection, MARCH1 is upregulated, which is helpful for the production of infectious virus titers by regulating iron levels [27]. Furthermore, MARCH1 inhibits IFN-I signaling pathway by promoting degradation of STING [28]. STING regulates the activity of TBK1 by directly interacting with TBK1 in innate immunity [19], whereas the TBK1-mediated mTOR signaling pathway plays an important role in tumor progression [22, 24]. However, it is unclear whether MARCH1 regulates tumor progression by targeting the TBK1-mTOR signaling pathway.

In this study, we showed that MARCH1 negatively regulated proliferation and migration of breast cancer cells by targeting the STING-TBK1-mTOR signaling pathway. MARCH1 interacted with TBK1 and promoted its K63-linked ubiquitination, thereby suppressing its interaction with mTOR. Taken together, these results indicate that the MARCH1-STING-TBK1-mTOR axis plays an important role in tumor progression of breast cancer cells.

## Materials and methods

### Cell culture and plasmids

The Sum159 and HEK293T cells were maintained in DMEM (Hyclone, USA) supplemented with 10% fetal bovine serum (OPCEL, China) and 1% penicillin- streptomycin (SparkJade, China). The MCF7 and MFM223 cells were maintained in RPMI1640 (Hyclone, USA) supplemented with 10% fetal bovine serum and 1% penicillin-streptomycin. All the cells were cultured at 37 °C in a humidified atmosphere containing 5% CO2. The cell lines were all monitored to detect mycoplasma contamination, that showed they were not contaminated. The cells were then transfected with the related plasmids and 1 mg/ml of PEI (YEASEN Biotech, China) according to the instructions of the manufacturer.

The related genes were amplified from HEK293T cDNA and then subcloned to pCDN3.1-flag, NBLV0051, MC-Myc-pCS2, or MC-HA-pCS2. The site directed mutagenesis of the related plasmids was constructed using a pair of complementary primers with the desired mutation.

### Antibodies and reagents

The antibodies and reagents used in the study were as follows: p-TBK1 S172 (CST, 5483S); TBK1 (Proteintech, 28397-1-AP); p-S6K1 T389 (ABclonal, AP1059); S6K1 (ABclonal, A2190); p-AKT S473 (ABclonal, AP0637); AKT1 (CST, 2938S); mTOR (CST, 2983S); MARCH1 (ImmunoWay, YT2642); DYKDDDDK-Tag (Abmart, M20008L); MYC-Tag (CST, 2276S); V5-Tag (Abclonal, AE017); HA (CST, 3724S); HA (Abclonal, AE036); GAPDH (Abmart, M20006L); β-actin (Abclonal, AC038); β-tublin (Abmart, M20005L); p-ULK1(S757) (CST, 6888); ULK1(Abclonal, A8529); SGK1(CUSABIO, CSB-PA021189LA01HU); p-SGK1(S422) (CUSABIO, CSB-PA050044); goat anti-Mouse IgG HRP (Abmart, M21001S); goat anti-rabbit IgG HRP (Abmart, M21002S); goat anti-mouse IgG AF 488 (Abmart, M21011M); goat anti-rabbit IgG AF 594 (Abmart, M21014M); goat anti-mouse IgG AF 594 (Abmart, M21013M); goat anti-rabbit IgG AF 488 (Abmart, M21012M); EGF (Solarbio, P00033); insulin (Solarbio, I8830); Rapamycin (Selleck, S1039) and H151 (MCE, HY-112693); MG132 (Millipore, 474790); Torin 1 (MCE, HY-13003); GSK8612 (MCE, HY-111941); and ZSTK474 (MCE, HY-50847).

### Establishment of stable knock-down cells

The shRNA sequences against the negative controls (NCs), MARCH1, or TBK1 were synthesized by Tsingke Biotech Co., Ltd and then ligated to the pLKO.1-TRC plasmid. The constructed shRNA plasmids were then transfected with psPAX2 and pMD2.G plasmids in HEK293T cells using 1 mg/ml of PEI (YEASEN Biotech, China) according to the manufacturer’s instructions. After transfection for 48 h, the cell supernatant of HEK293T was collected and filtered using a 0.45 μm filter to infect the related cell lines. After infection for 48 h, the cells were selected with puromycin (2 ug/ml for Sum159, 7 ug/ml for MCF7, and 1 ug/ml for HEK293T) until a single cell colony was formed. The sequences against NC, MARCH1, TBK1, and Rictor were synthesized as follows: TTCTCCGAACGTGTCACGT for NC, GCAAGATATCAACCATGTATT for MARCH1, GCAGAACGTAGATTAGCTTAT for TBK1, and CGTCGGAGTAACCAAAGATTA for Rictor.

### Western blotting

The cells were rinsed with phosphate buffered saline (PBS) and then lysed with 1 × SDS loading buffer and boiled at 100℃ for 10 min. The cell lysates were electrophoresed on SDS-PAGE gels to separate the target proteins and then transferred to polyvinylidene difluoride (PVDF) membranes (Cobetter, China). After blocking with 5% skimmed milk in tris-buffered saline with Tween 20 (TBST) at room temperature for 1 h, the PVDF membrane was sequential incubated with the related primary antibodies overnight at 4℃ and the corresponding secondary antibodies for 2 h at room temperature. The enhanced chemiluminescence (ECL) reaction was performed using a ECL chemiluminescence substrate kit (Biosharp, China). All the experiments were repeated three times.

### Immunoprecipitation

The HEK293T cells seeded in 100 mm cell culture dishes were co-transfected for 24 h with the corresponding plasmids to overexpress the target proteins. After washing with PBS, the cells were lysed with ice cold NETN buffer (20 mM Tris pH-8.0, 150 mM NaCl, 0.5% NP-40, and 1 mM EDTA) supplemented with an EDTA-free complete protease inhibitor cocktail (Selleck). After shaking for 30 min at 4℃, the protein samples were separated by centrifugation at 4℃ for 10 min at 15,000 g and then incubated with the corresponding beads for 6 h at 4℃. After washing three times with ice cold NETN buffer, the Flag-bead bound proteins were eluted with 1 × SDS loading buffer and then boiled for 5 min. The samples were separated and analyzed by Western blotting as described in the previous section. All the experiments were repeated three times.

For the denatured immunoprecipitation of ubiquitination, HEK 293 T cells were lysed with 1 × SDS loading buffer and boiled at 100℃ for 10 min. The cell lysates were then mixed into octuple ice cold NETN buffer and incubated with the corresponding beads for 6 h at 4℃. After washing three times with NETN buffer the beads were lysed with 1 × SDS loading buffer and boiled at 100℃ for 10 min.

### Insulin and EGF assay

For growth factor stimulation, the cells were starved with serum-free cell medium for 50 min and then re-stimulated with insulin or EGF for the indicated times. The final concentration of growth factors was kept at 100 nM for insulin and 25 ng/ml for EGF. Torin 1, GSK8612, H151, Rapamycin, and ZSTK474 were dissolved in dimethyl sulfoxide (DMSO) and added to the culture medium to a final concentration of 100 nM, 2 μM, 1 μM, 5 μM, and 100 nM, respectively. Torin 1, GSK8612, Rapamycin, and ZSTK474 were added 50 min prior to being lysed, while H151 was added 6 h prior to being lysed.

### Scratch wound healing assay

When the MCF7 cells reached 90 -100% confluence in the six-well cell culture plates, linear scratch wounds were made on the cell surface layer using a 10 μL pipette tip. The cells were washed twice with PBS to remove cellular debris and then supplemented with a fresh cell culture medium free of fetal bovine serum. The scratch wounds were imaged at 0, 6, 12, 24, and 48 h and the area of the scratch wounds was measured by Image J The data were analyzed statistically using Student’s t-test. All the experiments were repeated three times.

### Clonogenic cell survival assay

MCF7 cells were seeded in six-well cell culture plates with about 1000 cells per well and then cultured for 14 d in RPMI1640 with 10% fetal bovine serum. The cell culture medium was changed every three days. After cell colonies were observed, the cells were rinsed twice with PBS and then fixed with 4% paraformaldehyde for 10 min. After washing three times with PBS, the cells were stained with 1 ml of crystal violet nonahydrate for 10 min and the cells were then rinsed with PBS, dried, and imaged.

### Statistical analyses

**Fig. 1:**
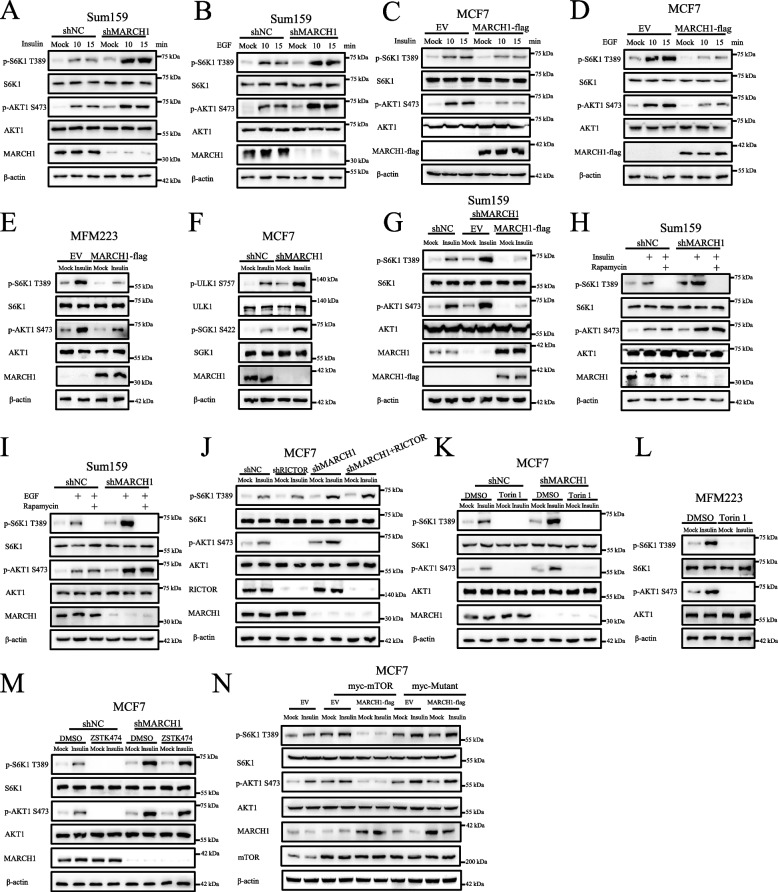
MARCH1 negatively regulates growth factor induced mTOR signaling pathway. **A**-**B** A deficiency of MARCH1 enhanced growth factor induced the mTOR signaling pathway. The stable knocked-down negative control (shNC) and MARCH1 (shMARCH1) Sum159 cells lines were starved of serum for 50 min and re-stimulated with 100 nM of insulin (**A**) or 25 ng/ml EGF (**B**) for 10 or 15 min. **C**-**D** MARCH1 overexpression attenuated the mTOR signaling pathway. MCF7 cells expressing EV or MARCH1-flag were starved of serum for 50 min and re-stimulated with insulin (**C**) or EGF (**D**) at the times indicated. **E** MARCH1 overexpression suppressed the mTOR signaling pathway in MFM223 cells. MFM223 cells expressing EV or MARCH1-flag were starved of serum for 50 min and re-stimulated with insulin (100 nM) for 15 min. **F** The mTOR signaling pathway was enhanced in MARCH1-deficient cells. shNC and shMARCH1 MCF7 cells were treated as described in **A**. **G** The rescue of MARCH1 in shMARCH1 cells suppressed the mTOR signaling pathway. shMARCH1 Sum159 cells were rescued with a shRNA-resistant MARCH1 plasmid. The cells were treated as described in **F**. **H**-**I** Rapamycin suppressed the enhancement of the mTORC1 signaling pathway. Stable shNC or shMARCH1 Sum159 cells were starved of serum for 50 min and re-stimulated with insulin (**H**) or EGF (**I**) for 15 min. Rapamycin (5 μM) was added to the cells for 50 min prior to lysis of the cells. **J** An absence of Rictor inhibited the MARCH1 deficiency induced by mTORC2 activation. The shNC, shRictor, shMARCH1, or shMARCH1 + Rictor MCF7 cells were treated as described in **F**. **K** Torin 1 inhibited the mTOR signaling pathway. shNC and shMARCH1 MCF7 cells were starved of serum for 50 min and re-stimulated with insulin (100 nM) for 15 min. DMSO or Torin 1 (100 nM) was added to the cell culture medium for 50 min prior to lysis of the cells. **L** Torin 1 suppressed the enhancement of the mTOR signaling pathway in MFM223 cells. MFM223 cells were starved of serum for 50 min and re-stimulated with insulin (100 nM) for 15 min. DMSO or Torin 1 (100 nM) was added to the cell culture medium for 50 min prior to lysis of the cells. **M** ZSTK474 showed little effect on the mTOR signaling pathway in MARCH1-deficient cells. The shNC and shMARCH1 MCF7 cells were starved of serum for 50 min and re-stimulated with insulin (100 nM) for 15 min. DMSO or ZSTK474 (100 nM) was added to cell culture medium for 50 min prior to lysis of the cells. **N** The overexpression of the mTOR mutant (mTOR-S2159D) prevented the attenuation of the mTOR signaling pathway induced by overexpression of MARCH1. MCF7 cells were overexpressed with the indicated plasmids and then starved of serum for 50 min, followed by stimulation with insulin (100 nM) for 15 min. All the experiments were repeated three times

**Fig. 2:**
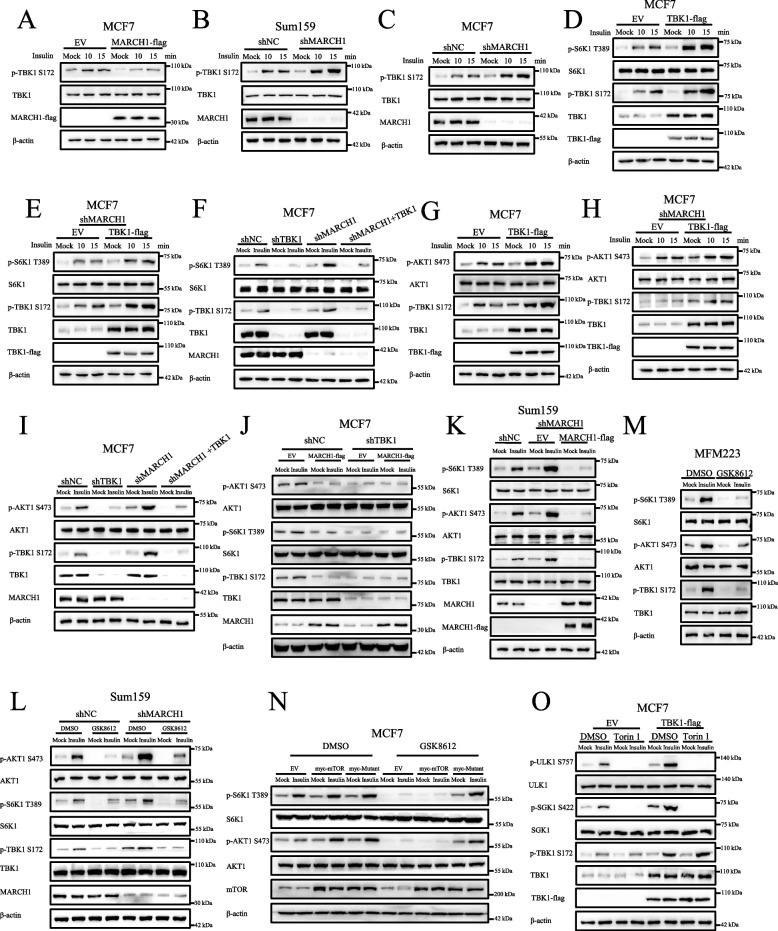
TBK1 acts as the downstream protein of MARCH1 to regulate the mTOR signaling pathway. **A** MARCH1 overexpression attenuated the phosphorylation of TBK1 at S172. MCF7 cells overexpressing MARCH1-flag were starved of serum for 50 min and re-stimulated with insulin (100 nM) for 10 or 15 min. **B**-**C** A deficiency of MARCH1 enhanced the phosphorylation of TBK1 at S172. The shNC and shMARCH1 Sum159 (**B**) or MCF7 (**C**) cells were treated as described in **A**. **D**-**E** TBK1 overexpression enhanced mTORC1 activity. The wild-type (**D**) or shMARCH1 (**E**) MCF7 cells expressing EV or TBK1-flag were treated as described in **A**. **F** A deficiency of TBK1 suppressed the enhanced mTORC1 activity in MARCH1-deficient cells. The shNC, shTBK1, shMARCH1, and shMARCH1 + TBK1 MCF7 cells were starved of serum for 50 min and re-stimulated with insulin (100 nM) for 15 min. **G**-**H** TBK1 overexpression enhanced the mTORC2 signaling pathway. Wild-type (**G**) or MARCH1-deficient MCF7 cells (**H**) transfected with TBK1-flag were treated as described in **A**. **I** A deficiency of TBK1 suppressed the enhanced mTORC2 signaling pathway in MARCH1-deficient cells. The shNC, shTBK1, shMARCH1, and shMARCH1 + TBK1 MCF7 cells were treated as described in **F**. **J** The attenuated mTOR signaling pathway in TBK1-deficient cells was not affected by MARCH1 overexpression. The shNC and shTBK1 MCF7 cells expressing MARCH1-flag were treated as described in **F**. **K** The rescue of MARCH1 in shMARCH1 cells inhibited the phosphorylation of TBK1 at S172 induced by insulin. The shMARCH1 Sum159 cells were rescued with shRNA-resistant MARCH1 plasmid. The cells were then treated as described in **F**. **L** GSK8612 suppressed the enhanced mTOR signaling pathway in MARCH1-deficient cells. The shNC and shMARCH1 Sum159 cells were starved of serum for 50 min and re-stimulated with insulin for 10 and 15 min. DMSO or GSK8612 (2 μM) was added to cells for 50 min prior to them being lysed. **M** GSK8612 attenuated the mTOR signaling pathway in MFM223 cells. The wild type MFM223 cells were starved of serum for 50 min and re-stimulated with insulin for 15 min. DMSO or GSK8612 (2 μM) was added to the cells for 50 min prior to them being lysed. **N** The overexpression of the mTOR mutant (mTOR-S2159D) prevented the attenuation of the mTOR signaling pathway induced by GSK8612. MCF7 cells expressing the indicated proteins were treated as described in **L**. **O** Torin 1 aborted the enhancement of the mTOR signaling pathway in cells overexpressing TBK1. MCF7 cells expressing EV or TBK1-flag were starved of serum for 50 min and re-stimulated with insulin (100 nM) for 15 min. Torin 1 (100 nM) was added to the cells for 50 min prior to them being lysed. All the experiments were repeated three times

**Fig. 3:**
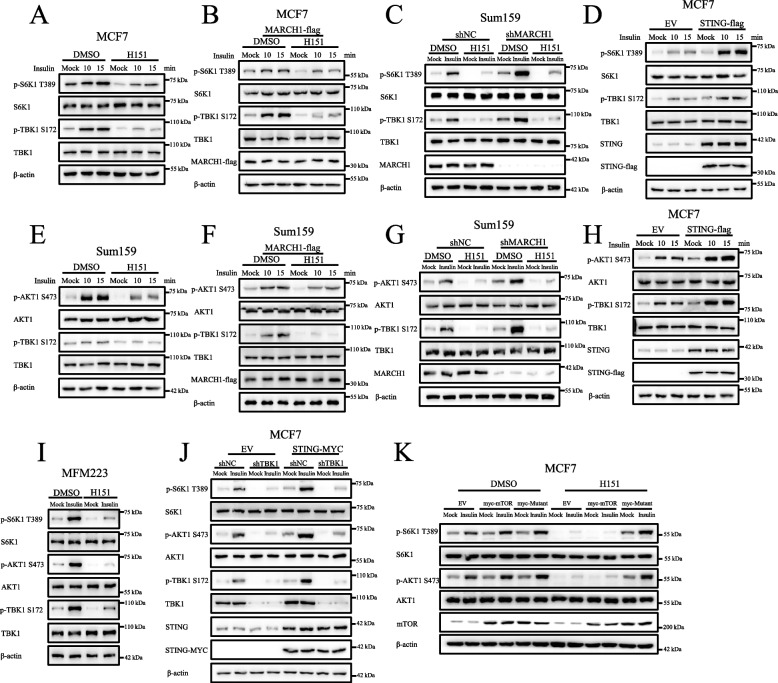
STING mediates signal transduction from MARCH1 to TBK1. **A**-**B** The STING inhibitor H151 suppressed the mTORC1 signaling pathway. Wild-type (**A**), or MARCH1 overexpressed (**B**) MCF7 cells were starved of serum for 50 min and re-stimulated with insulin (100 nM) for 10 or 15 min. DMSO or H151 (1 μM) was added to the medium for 6 h prior to the cells being lysed. **C** H151 attenuated enhancement of the mTORC1 signaling pathway in MARCH1-deficient cells. The shNC and shMARCH1 Sum159 cells were starved of serum for 50 min and re-stimulated with insulin (100 nM) for 15 min. DMSO or H151 (1 μM) was added to medium for 6 h prior to the cells being lysed. **D** The overexpression of STING enhanced the mTORC1 signaling pathway. MCF7 cells expressing EV or STING-flag were starved of serum for 50 min and re-stimulated with insulin (100 nM) for 10 or 15 min. **E**-**G** H151 suppressed the mTORC2 signaling pathway. Wild-type (**E**), MARCH1-overexpressed (**F**) or MARCH1-deficient (**G**) Sum159 cells were treated as described in **A**. **H** STING overexpression activated the mTORC2 signaling pathway. MCF7 cells expressing EV or STING-flag were treated as described in **D**. **I** H151 attenuated the mTOR signaling pathway. The wild type MFM223 cells were starved of serum for 50 min and re-stimulated with insulin (100 nM) for 15 min. DMSO or H151 (1 μM) was added to medium for 6 h prior to the cells being lysed. **J** A deficiency of TBK1 attenuated the enhancement of the mTOR signaling pathway induced by STING overexpression. The shNC and shTBK1 MCF7 cells expressing EV or STING-MYC were starved of serum for 50 min and re-stimulated with insulin (100 nM) for 15 min. **K** The overexpression of the mTOR mutants (mTOR-S2159D, myc-Mutant) prevented the attenuation of the mTOR signaling pathway induced by H151. MCF7 cells expressing the indicated plasmids were treated as described in **C**. All the experiments were repeated three times

**Fig. 4:**
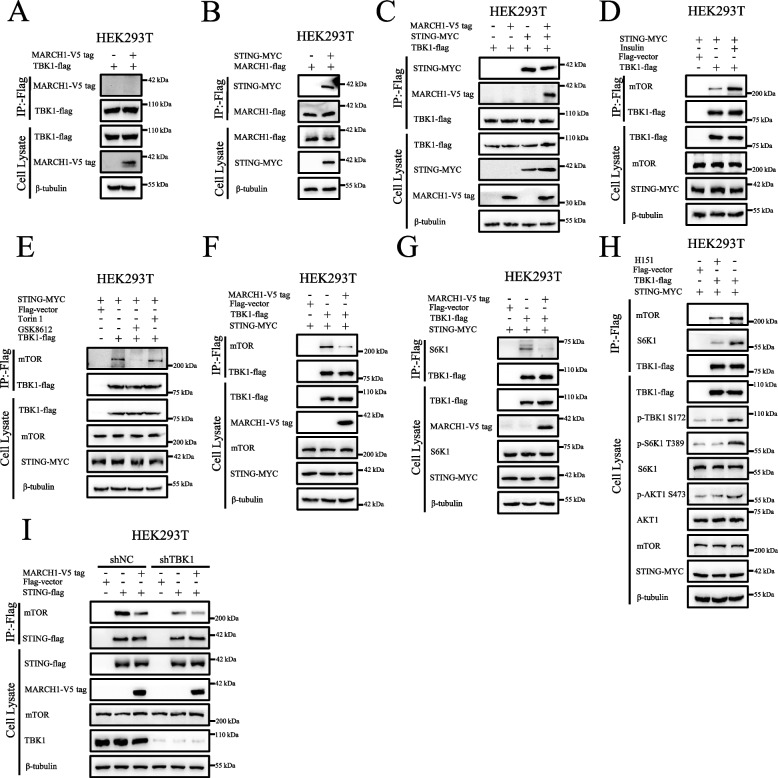
STING mediates the indirect interaction between MARCH1 and TBK1. **A** MARCH1 did not interact with TBK1. HEK293T cells expressing the indicated proteins were lysed with NETN lysis buffer and then immunoprecipitated with flag beads to detect these proteins. **B** MARCH1 interacted with STING. HEK293T cells transfected with the indicated plasmids were lysed with NETN lysis buffer and then immunoprecipitated with flag beads to detect the indicated proteins. 10 μM MG132 was added to the cells for 12 h prior to them being lysed. **C** MARCH1 interacted with the TBK1 in the presence of STING. HEK293T cells expressing the indicated proteins were treated as described in **B**. **D** The interaction between TBK1 and mTOR was enhanced in response to insulin stimulation. HEK293T cells expressing the indicated proteins were starved of serum for 16 h and re-stimulated with insulin (100 nM) for 15 min. The collected cells were treated as described in **A**. **E** GSK8612 attenuated the interaction between TBK1 and mTOR. HEK293T cells expressing the indicated proteins were incubated with GSK8612 (2 μM) or Torin 1 (100 nM) for 50 min prior to the cells being lysed. **F** MARCH1 decreased the interaction between TBK1 and mTOR. HEK293T cells expressing the indicated proteins were treated as described in **B**. **G** MARCH1 decreased the interaction between TBK1 and S6K1. HEK293T cells expressing the indicated proteins were treated as described in **B**. **H** H151 decreased the interaction of TBK with mTOR or S6K1. HEK293T cells expressing EV or TBK1-flag were treated with H151 (1 μM) for 6 h, and then subjected to immunoprecipitation with flag beads to detect the indicated proteins. **I** The interaction between STING and mTOR was weakened by a lack of TBK1 or overexpression of MARCH1. The shNC and shTBK1 HEK293T cells expressing the indicated proteins were treated as described in **B**. All the experiments were repeated three times

**Fig. 5:**
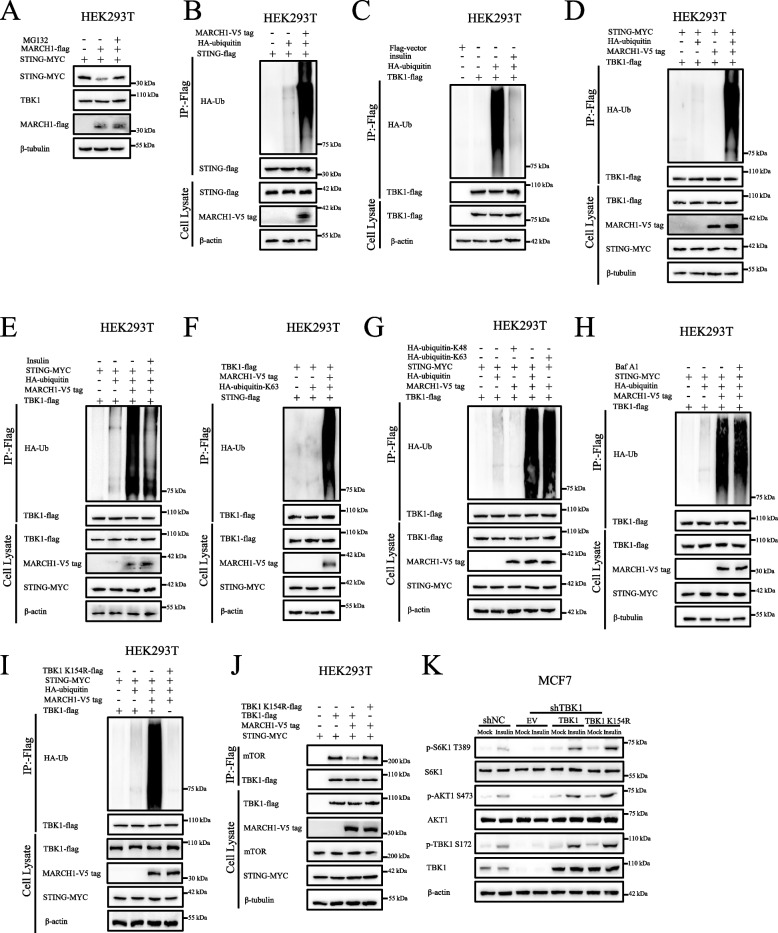
MARCH1 promotes the ubiquitination of TBK1. **A** MARCH1 promoted the degradation of STING. HEK293T cells expressing the indicated proteins were incubated with DMSO or MG132 (10 μM) for 12 h. **B** MARCH1 promoted the ubiquitination of STING. HEK293T cells transfected with the indicated plasmids were lysed with 1 × SDS loading and then subjected to denatured immunoprecipitation with flag beads to detect the indicated proteins. 10 μM MG132 was added to cells for 12 h prior to the cells being lysed. **C** The ubiquitination of TBK1 was attenuated in response to insulin stimulation. HEK293T cells transfected with the indicated plasmids were starved of serum for 16 h and re-stimulated with insulin for 15 min. The collected cells were lysed with 1 × SDS loading and then subjected to denatured immunoprecipitation with flag beads to detect the indicated proteins. **D** MARCH1 promoted the ubiquitination of TBK1. HEK293T cells expressing the indicated proteins were treated as described in **B**. **E** The ubiquitination of TBK1 mediated by MARCH1 was attenuated in response to insulin treatment. HEK293T cells expressing the indicated proteins were starved of serum for 16 h and re-stimulated with insulin for 15 min. MG132 (10 μM) was added to cells for 12 h prior to the cells being lysed. **F** MARCH1 promoted the K63-linked ubiquitination of TBK1. HEK293T cells expressed the indicated proteins treated as described in **B**. **G** MARCH1 mediated the K63-linked ubiquitination of TBK1. HEK293T cells expressed the indicated proteins treated as described in **B**. **H** The ubiquitination of TBK1 induced by MARCH1 was not mediated by an autophagy process. HEK293T cells expressing the indicated proteins were lysed with 1 × SDS loading and then subjected to denatured immunoprecipitation with flag beads to detect the indicated proteins. DMSO or Bafilomycin A1 (Baf A1, 0.2 μM) was added to the cells for 12 h prior to them being lysed. **I** TBK1 K154R aborted its ubiquitination mediated by MARCH1. HEK293T cells expressing the indicated proteins were treated as described in **B**. **J** The decreased interaction between TBK1 and mTOR induced by MARCH1 overexpression was restored by TBK1 K154R. The collected cells were lysed with NETN lysis buffer and then immunoprecipitated with flag beads to detect the indicated proteins. **K** TBK1 K154R enhanced the mTOR signaling pathway. The shNC and shTBK1 MCF7 cells were rescued with shRNA-resistant TBK1 or TBK1 K154R plasmids. The cells were then lysed to perform Western blotting

The gray value of each blot from p-AKT1, p-S6K1, p-TBK1, p-ULK1, and p-SGK1 was calculated with Image J (FIJI) and normalized to the loading control (β-actin for Figs. 1, 2, 3 and 5K and β- tubulin for Figs. 4 and 5A, J). The normalized data from Figs. 1, 2, 3 and 5K were then re-normalized to the value obtained from the first line of each blot. Similarly, the normalized data from Fig. 4B-I was re-normalized to the value obtained from the second or third lane of each blot.

Student’s t-test was used to compare two experimental conditions (Figs. S1E-N, S2F, I-O, S3C, S3G, S3I-K, S4A-C, S4E-G, S4I, 6B, D and F). For comparison of more than two conditions, a one-way ANOVA test was applied (Figs. S1A-D, S2A-E, S2G-H, S3A-B, S3D-F, S3H, S4D, S4H, and S4J-K).

**Fig. 6:**
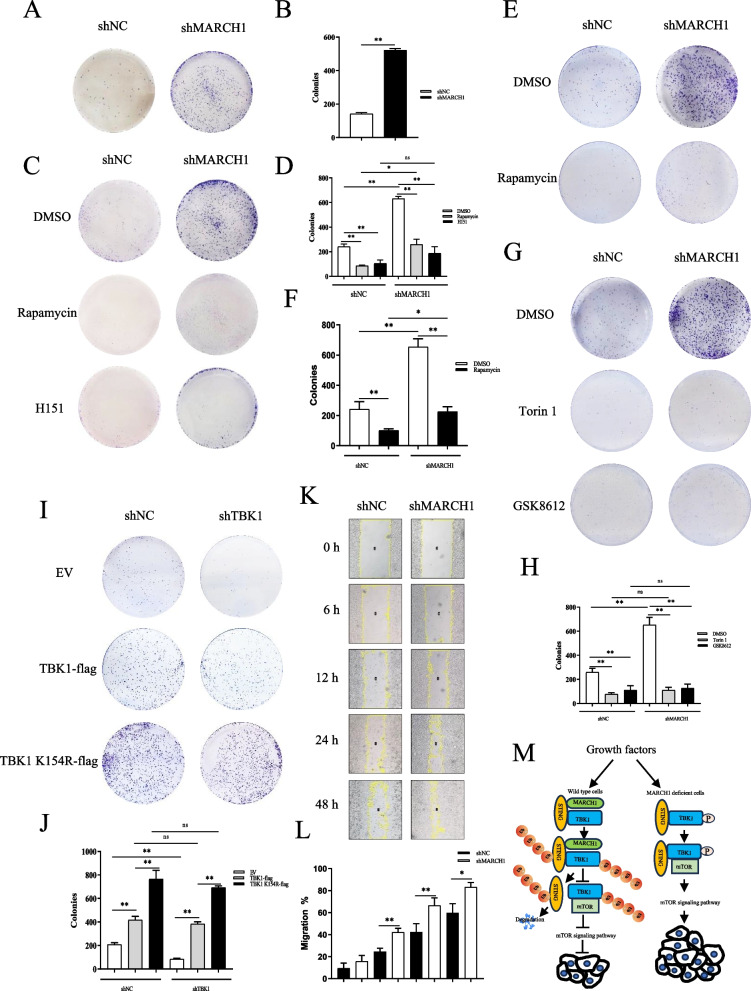
A deficiency of MARCH1 enhanced breast cancer cell proliferation and migration. **A**-**B** A deficiency of MARCH1 promoted the proliferation of breast cancer cells. Stable shNC or shMARCH1 MCF7 cells were seeded in six-well plates and cultured for 14 d. The cell culture medium was changed every 3 d. The cells were fixed with 4% PFA and stained with crystal violet solution (**A**). The number of colonies was counted and analyzed statistically using GraphPad Prism 8.0 (**B**). **C**-**D** H151 and rapamycin suppressed faster cell proliferation in MARCH1-deficient breast cancer cells. Stable shNC or shMARCH1 MCF7 cells seeded in six-well plates were cultured with DMSO, H151 (1 μM) or rapamycin (5 μM) for 14 d (**C**). The number of colonies was counted and analyzed statistically using GraphPad Prism 8.0 (**D**). **E**-**F** Rapamycin suppressed the faster proliferation of MARCH1-deficient breast cancer cells. Stable shNC or shMARCH1 MCF7 cells were seeded in six-well plates and incubated with DMSO, or rapamycin (100 nM) for 14 d (**E**). The number of colonies was counted and analyzed statistically using GraphPad Prism 8.0 (**F**). **G**-**H** Torin 1 and GSK8612 suppressed the faster proliferation of MARCH1-deficient breast cancer cells. Stable shNC or shMARCH1 MCF7 cells were seeded in six-well plates and incubated with DMSO, Torin 1 (100 nM) or GSK8612 (2 μM) for 14 d (**G**). The number of colonies was counted and analyzed statistically using GraphPad Prism 8.0 (**H**). **I**-**J** TBK1 K154R promoted the faster cell proliferation. shNC and shTBK1 MCF7 cells were rescued with EV, shRNA resistant TBK1-flag or TBK1 K154R-flag plasmids and cultured in six-well plates for 14 d (**I**). The number of colonies was counted and analyzed statistically using GraphPad Prism 8.0 (**J**). **K**-**L** A deficiency of MARCH1 enhanced the migration of breast cancer cells. Stable shNC or shMARCH1 MCF7 cells were scratched, washed twice with PBS and then cultured further with medium free of FBS. The images were acquired at 0, 6, 12, 24 and 48 h after scratching (**K**). The areas of migration were calculated using Image J and analyzed statistically using GraphPad Prism 8.0 (**L**). **M** A schematic showing the mechanism by which MARCH1 regulates mTOR signaling pathway via targeting TBK1. All the experiments were repeated three times. The error bars indicate the mean (SD). *p < 0.05, **p < 0.01

All the statistical analyses and preparation of the columns in the figures were performed using Graphpad Prism 8.0.

## Results

### MARCH1 negatively regulates the mTOR signaling pathway

To determine the role of MARCH1 in the mTOR signaling pathway, we constructed the MARCH1 knocked-down breast cancer cell line. We showed that a deficiency of MARCH1 enhanced the phosphorylation level of S6K1 at T389 (p-S6K1) and AKT1 at S473 (p-AKT1) in response to insulin stimulation (Figs. 1A and S1A). Similar results were obtained by stimulation with EGF (Figs. 1B and S1B). However, overexpression of MARCH1 decreased insulin or EGF-induced p-S6K1 and p-AKT1 (Figs. 1C-D and S1C-1D). Moreover, MARCH1 overexpression suppressed the mTOR signaling pathway in MFM223 cells (Figs. 1E and S1E),which express low level of endogenous MARCH1. A deficiency of MARCH1 also enhanced phosphorylation of ULK1 at S757 and SGK1 at S422 (Figs. 1F and S1F), which are the direct downstream proteins of mTORC1 and mTORC2, respectively. To determine whether the enhancement of the mTOR signaling pathway in MARCH1 deficient cells was due to a lack of MARCH1, we rescued shRNA-resistant MARCH1 plasmids in MARCH1-deficient cells. The results showed that enhancement of the mTOR signaling pathway in MARCH1 deficient cells was attenuated by overexpression of MARCH1 (Figs. 1G and S1G). These results demonstrated that MARCH1 negatively regulated growth factor induction of the mTOR signaling pathway.

To further confirm whether MARCH1 acted upstream of the mTOR signaling pathway, we first incubated MARCH1-deficient cells with Rapamycin, an inhibitor of mTORC1. In response to growth factor stimulation, Rapamycin inhibited the enhanced p-S6K1 in the MARCH1-deficient cells, whereas it had little effect on p-AKT1 (Figs. 1H-I and S1H-1I). In addition, a lack of Rictor, an important component of mTORC2, only affected p-AKT1 in the MARCH1-deficient cells (Figs. 1J and S1J). Torin 1, an ATP-competitive mTOR inhibitor with preferential activity against mTOR compared to PI3K [29], significantly inhibited the mTOR signaling pathway in MARCH1-deficient cells (Figs. 1K and S1K). Torin 1 also suppressed the mTOR signaling pathway in MFM223 cells (Figs. 1L and S1L). To determine the role of PI3K in the MARCH1-mediated mTOR signaling pathway, we treated cells with ZSTK474, an ATP-competitive inhibitor of PI3K and a weak mTOR inhibitor [30]. ZSTK474 treatment did not prevent the enhancement of the mTOR signaling pathway in MARCH1-deficient cells (Figs. 1M and S1M), which indicated that the MARCH1-mediated mTOR signaling pathway did not involve PI3K. Importantly, the overexpression of the mTOR phosphomimetic mutants (mTOR-S2159D, myc-Mutant) restored the attenuation of the mTOR signaling pathway induced by MARCH1 overexpression (Figs. 1N and S1N). These results therefore suggest that MARCH1 acts upstream of the mTOR signaling pathway.

### TBK1 acts as the downstream protein of MARCH1 to regulate the mTOR signaling pathway

Considering that TBK1 regulates the mTOR signaling pathway by directly interacting with mTOR [22, 23], we speculated that MARCH1 may regulate the mTOR signaling pathway by targeting TBK1. To prove this hypothesis, we examined the effects of MARCH1 on the phosphorylation of TBK1 at the Ser172 site (p-TBK1 S172), the marker of TBK1 activation. As expected, insulin stimulation enhanced p-TBK1 S172 (Fig. 2A and S2A), which was markedly attenuated by overexpression of MARCH1 (Figs. 2A and S2A). As expected, MARCH1 deficiency greatly enhanced p-TBK1 S172 (Figs. 2B-C and S2B-2C). These results suggested that MARCH1 may regulate the mTOR signaling pathway by targeting TBK1.

To further determine the role of TBK1 in the MARCH1-mediated mTOR signaling pathway, we first examined its effect on the mTORC1 signaling pathway. Overexpression of TBK1 enhanced the p-S6K1 in response to insulin stimulation (Figs. 2D-E and S2D-2E). Correspondingly, a lack of TBK1 suppressed the enhancement of p-S6K1 in MARCH1-deficient cells (Figs. 2F and S2F). In addition to promoting the mTORC1 signaling pathway, TBK1 overexpression also enhanced the mTORC2 signaling pathway (Figs. 2G-H and S2G-2H). Correspondingly, a lack of TBK1 attenuated the enhancement of p-AKT1 in MARCH1-deficient cells (Figs. 2I and S2I). Furthermore, the decreased activity of the mTOR signaling pathway was not further attenuated by MARCH1 overexpression in TBK1-deficient cells (Figs. 2J and S2J). The rescue of shRNA-resistant MARCH1 plasmids in MARCH1-deficient cells suppressed the activation of p-TBK1 S172 (Figs. 2K and S2K). In addition, GSK8612, an inhibitor of TBK1 [31], clearly decreased the enhancement of the mTOR signaling pathway in MARCH1-deficient cells (Figs. 2L and S2L). GSK8612 also suppressed the mTOR signaling pathway in MFM223 cells (Figs. 2M and S2M). Importantly, GSK8612 did not attenuate the mTOR signaling pathway by overexpression of mTOR phosphomimetic mutants (mTOR-S2159D, myc-Mutant) (Figs. 2N and S2N). This finding further confirmed the upstream role of TBK1 in the mTOR signaling pathway. To further determine the role of TBK1 in the mTOR signaling pathway, we detected phosphorylation of ULK1 (S757) and SGK1 (S422) in response to the mTOR inhibitor, Torin 1. The results showed that Torin 1 clearly attenuated the enhancement of p-ULK1 and p-SGK1 induced by overexpression of TBK1 (Figs. 2O and S2O). Based on the results above, we consider that TBK1 acts as a downstream protein of MARCH1 to regulate the mTOR signaling pathway.

### STING mediates the signal transduction from MARCH1 to TBK1

Since TBK1 activation requires STING [21, 32], we then examined whether STING was involved in the growth factor-mediated MARCH1-TBK1-mTOR axis. H151, an inhibitor of STING activation, not only attenuated p-TBK1 S172 but also attenuated p-S6K1 in response to insulin stimulation (Figs. 3A and S3A). Moreover, H151 not only decreased p-S6K1 in MARCH1 overexpressed cells (Figs. 3B and S3B), but also decreased p-S6K1 in MARCH1-deficient cells (Figs. 3C and S3C). However, the overexpression of STING enhanced insulin-induced p-S6K1 (Figs. 3D and S3D). These results indicated that the activity of STING plays an important role in the growth factor- mediated MARCH1-TBK1-mTOR signaling pathway.

In addition to its role in mTORC1, STING also regulated the mTORC2 signaling pathway. We showed that H151 decreased p-AKT1 not only in wide type cells (Figs. 3E and S3E) but also in cells overexpressing MARCH1 (Figs. 3F and S3F). The increased activity of p-AKT1 in MARCH1-deficient cells was attenuated by H151 treatment (Figs. 3G and S3G). However, overexpression of STING enhanced insulin-induced activity of p-AKT1 (Figs. 3H and S3H). Importantly, H151 also attenuated the mTOR signaling pathway in MFM223 cells (Figs. 3I and S3I). Based on these results, we concluded that STING acts as a bridge to mediate signaling transduction from MARCH1 to TBK1 to regulate the mTOR signaling pathway.

To further determine the role of STING in the TBK1-mTOR signaling pathway, we overexpressed STING in TBK1-deficient cells. The results showed that enhancement of the mTOR signaling pathway induced by STING overexpression was attenuated due to a lack of TBK1 (Figs. 3J and S3J). Moreover, H151 treatment had little effect on mTOR phosphomimetic mutants (mTOR-S2159D, myc-Mutant) induced the increased activity of mTOR signaling pathway (Figs. 3K and S3K). This finding further emphasized the important role of STING activity in the mTOR signaling pathway.

### STING mediates the interaction between MARCH1 and TBK1

To determine how STING mediated signaling transduction from MARCH1 to TBK1, we detected whether these three proteins interacted with each other. The results of the immunoprecipitation assay showed that MARCH1 did not interact with TBK1 in HEK293T cells (Fig. 4A). This may have been due to the deficient expression of STING in the HEK293T cells [33, 34]. MARCH1 was shown to interact with STING (Figs. 4B and S4A), a finding consistent with that of a previous report [28]. Moreover, MARCH1 interacted with TBK1 in the presence of STING (Figs. 4C and S4B). Therefore, STING acted as a crucial medium for the interaction between MARCH1 and TBK1.

Considering that TBK1 interacts with mTOR to regulate the mTOR signaling pathway [22], we decided to determine whether MARCH1 affected the interaction between TBK1 and mTOR. We showed that TBK1 interacted with mTOR (Fig. 4D), and this interaction was enhanced in response to insulin stimulation (Figs. 4D and S4C). However, this interaction was attenuated in response to the TBK1 inhibitor, GSK8612 (Figs. 4E and S4D), although the interaction was little affected by treatment with the mTOR inhibitor, Torin 1 (Figs. 4E and S4D). In addition, the interaction was markedly attenuated by overexpression of MARCH1 (Figs. 4F and S4E). TBK1 also interacted with S6K1 (Fig. 4G), a finding consistent with that of a previous report [22]. However, this interaction was also decreased by overexpression of MARCH1 (Figs. 4G and S4F). H151, an inhibitor of STING activity, also decreased the interaction between TBK1 and mTOR or S6K1 (Figs. 4H and S4G). As shown in Fig. 4I, STING interacted with mTOR. However, this interaction was attenuated by a lack of TBK1 or overexpression of MARCH1 (Figs. 4I and S4H). This indicated that the interaction between STING and mTOR was mediated by TBK1. Taken together, these results suggested that the interaction between TBK1 and mTOR was regulated by MARCH1 and STING.

### MARCH1 promotes the ubiquitination of TBK1

As one of the membrane-bound E3 ubiquitin ligases, MARCH1 overexpression promoted ubiquitin proteasome-mediated degradation of STING (Figs. 5A-B and S4I), a finding consistent with those of a previous report [28]. However, overexpression of MARCH1 did not affect the protein level of TBK1 (Fig. 5A). We then investigated whether MARCH1 could ubiquitinate TBK1. Considering that HEK293T cells respond to stimulation by growth factors [35], we detected the ubiquitination of TBK1 in response to insulin stimulation in HEK293T cells. Following serum starvation, TBK1 underwent ubiquitination (Fig. 5C), which was attenuated in response to insulin stimulation (Fig. 5C). Moreover, the ubiquitination of TBK1 was enhanced greatly by overexpression of MARCH1 (Fig. 5D), which could also be decreased in response to insulin stimulation (Fig. 5E). Moreover, TBK1 primarily underwent K63-linked ubiquitination mediated by MARCH1 (Figs. 5F-G). In addition, this ubiquitination mediated by MARCH1 was not via an autophagy process (Fig. 5H). To identify the ubiquitinated site of TBK1, we constructed different mutation plasmids by mutating lysine (K) of TBK1 to arginine (R) and showed that the mutation of TBK1 K154 to R (TBK1 K154R) aborted the MARCH1-mediated ubiquitination of TBK1 (Fig. 5I). Correspondingly, TBK1 K154R not only restored its decreased interaction with mTOR (Figs. 5J and S4J), but also enhanced the activity of the mTOR signaling pathway (Figs. 5K and S4K). Based on these results we concluded that MARCH1 promoted the k63-linked ubiquitination of TBK1.

### MARCH1 deficiency promotes faster proliferation and migration of breast cancer cells

To explore the role of MARCH1 in the proliferation of cancer cells, we performed a clonogenic cell survival assay using MARCH1-deficient MCF7 cells. As shown in Fig. 6A-B, a deficiency of MARCH1 clearly promoted proliferation of MCF7 cells, whereas H151 or rapamycin treatment markedly decreased the faster proliferation of MARCH1-deficient MCF7 cells (Fig. 6C-F). To determine the role of TBK1 and mTOR in cell proliferation, we treated MARCH1-deficient cells with the TBK1 inhibitor, GSK8612 or the mTOR inhibitor, Torin 1. The results showed that treatment with GSK8612 or Torin 1 obviously attenuated the faster proliferation in MARCH1-deficient cells (Fig. 6G-H). Moreover, TBK1 K154R overexpression also promoted faster proliferation in breast cancer cells, which further confirmed the important role of TBK1 K154 (Fig. 6I-J). To further determine the effect of MARCH1 on the migration of tumor cells, we performed a scratch wound healing assay. The results showed that the MARCH1 deficient cells migrated faster than wild type MCF7 cells (Fig. 6K-L). These results confirmed the important role of MARCH1 in the proliferation and migration of breast cancer cells.

## Discussion

As an important hub of metabolic regulation, mTOR signaling, including mTORC1 and mTORC2, needs to be fine-tuned. In this study, we showed that MARCH1 can simultaneously regulate the mTORC1 and mTORC2 signaling pathways by targeting TBK1. In response to growth factor stimulation, MARCH1 interacts with STING/TBK1 and promotes their ubiquitination. Subsequently, the ubiquitinated TBK1 decreases its interaction with mTOR and attenuates mTOR signaling pathway, thus inhibiting the proliferation of breast cancer cells (Fig. 6M). However, the lack of MARCH1 greatly promote the proliferation of breast cancer cells by enhancing mTOR signaling pathway (Fig. 6M).

As one member of the MARCH family, MARCH1 can regulate immunity by ubiquitinating some downstream proteins, such as CD86, MHC-II, and STING [26, 28, 36]. In addition, MARCH1 also regulates tumor progression. Su et al. [37] reported that MARCH1 suppressed bladder cancer growth, while Xu et al. [38] demonstrated that MARCH1 expression was decreased significantly in colon adenocarcinoma (COAD), lung adenocarcinoma, lung squamous cell carcinoma (LUSC), prostate adenocarcinoma (PAAD), and rectum adenocarcinoma (READ). Moreover, high expression of MARCH1 was always linked with better overall survival in lower grade glioma (LGG), lung adenocarcinoma, and skin cutaneous melanoma (SKCM) [38]. In the current study, we revealed the role of MARCH1 in tumor progression of breast cancer. MARCH1 interacts with TBK1 in the presence of STING and promotes the ubiquitination of TBK1 to negatively regulate the mTOR signaling pathway. Importantly, a deficiency of MARCH1 promotes the migration and the colony formation of breast cancer cells, which can be blocked by H151 (STING inhibitor), GSK8612 (TBK1 inhibitor), or Torin 1 (mTOR inhibitor). Therefore, MARCH1 may act as tumor suppressor gene in some cancers.

Since acting an important role in diverse cellular processes, such as immunity and autophagy [16, 39–41], the activity of TBK1 needs to be fine-tuned. The activity of TBK1 can be regulated by many post-translational modifications, such as phosphorylation and ubiquitination [42–45]. TBK1 can be auto-phosphorylated at S172 [18], which is recognized as active status. In addition, TBK1 S172 can also be phosphorylated by ULK1 and IKKβ [46, 47],thus regulating its activity. Besides, TBK1 activity can also be regulated by its ubiquitination at different sites. Wang et al. [48] reported that ubiquitination of TBK1 at K69 positively regulated its activity in response to a RNA virus infection. Similarly, Tu et al. [49] showed that the activity of TBK1 was positively regulated by its ubiquitination at the K30 and K401 sites. Moreover, TBK1 activity can be positively or negatively regulated by ubiquitination at the K670 site [50]. The current study showed that ubiquitination of TBK1 at the K154 site negatively regulated its activity in the growth factor-induced mTOR signaling pathway. However, Wang et al. [48] reported that ubiquitination at the K154 site promoted its activity in RNA virus-mediated innate immunity. Similarly, Lin et al. [50] reported that ubiquitination of TBK1 at the K670 site by different E3 ubiquitin ligases had the opposite effect on activity. Since the enhanced activity of TBK1 (TBK1 K154R) promotes the proliferation of breast cancer cells by targeting the mTOR signaling pathway, it is possible that ubiquitination of TBK1 at different sites may also regulate this proliferation by affecting its activity. However, the effects of TBK1 ubiquitination at different sites on the proliferation of breast cancer cells may depend on the E3 ubiquitin ligases which mediate the reaction.

As a serine/threonine kinase, TBK1 plays an important role in the mTOR signaling pathway. TBK1 activates the pathway by directly interacting with and phosphorylating mTOR on Ser2159 [22, 23] and activating mTORC1 by direct phosphorylation of AKT1 [51, 52]. In addition, TBK1 interacts with S6K to regulate the mTORC1 pathway [24]. However, it remains unknown how TBK1 mediation of the mTOR signaling pathway is regulated. We showed that MARCH1 promotes K63-linked ubiquitination of TBK1 at K154 site to regulate the mTOR signaling pathway, while MARCH1 overexpression attenuated the interaction between TBK1 and mTOR, resulting in reduced activity of the pathway. However, TBK1 K154R restored this decreased interaction with mTOR induced by overexpression of MARCH1. Importantly, the TBK1 K154R mutation obviously promoted the proliferation of breast cancer cells. Taken together these findings indicate that TBK1 has a positive role in the regulation of the mTOR signaling pathway. However, Kim et al. [53] reported that TBK1 inhibited the mTOR signaling pathway by interacting with mTOR in prostate cancer cells. There is also evidence in mice that TBK1 mediates inhibition of mTOR [54, 55]. These findings suggest that the different roles of TBK1 in mTOR signaling pathway may depend on the tissue-type.

As important proteins in innate immunity, STING and TBK1 play important roles in tumor progression. Activation of STING increases the presentation of tumor-associated antigens to CD8^+^ T cells by activating DCs [56]. In addition to promoting the trafficking and infiltration of T cells to tumors [57, 58], activation of STING also plays an important role in the recognition and killing of cancer cells by T cells [59, 60]. However, An et al. [61] reported that TBK1 had a negative role in immune infiltration of immune cells other than CD4^+^ T cells in pan cancers. In addition, high expression of TBK1 in tissues from hepatocellular carcinomas were shown to be associated with reduced tumor-infiltrating CD8^+^ T-cells and increased levels of immunosuppressive markers [62]. There is evidence from animal models of cancer immunotherapy that the deletion of TBK1 in dendritic cells causes T cell activation, subsequently enhancing antitumor immunity [63]. STING and TBK1 also have important roles in cancer cells. Activation of STING directly triggers cancer cell death in malignant B cells [64], while in triple-negative breast cancer, STING has been shown to have anti-tumor effects by promoting the type-I IFN signaling pathway [65]. In contrast, STING has been reported to promote the survival of breast cancer cells by enhancing DNA damage response and the activity of the IL-6-STAT3 survival pathway [66–68]. STING activation can also facilitate cancer metastasis by producing inflammatory cytokines [69], while intracellular STING inactivation sensitizes breast cancer cells to genotoxic agents [70]. These findings therefore indicate that high expression of STING is associated with an increased risk of relapse in breast cancer patients receiving adjuvant chemotherapy [68]. Similarly, increased TBK1 expression and/or aberrant TBK1 activity have been reported in many types of cancers, such as breast cancer [71], which may be due to the high expression of TBK1 promoting viability, proliferation, migration, and invasion of cancer cells through activation of the mTOR signaling pathway [72]. Barbie et al. [73] reported that TBK1 promotes cell survival and proliferation in some KRAS mutant cells. Therefore, TBK1 inhibition sensitizes breast cancer cells to tamoxifen-induced cell death [74]. In this study, we identified that the STING-TBK1-mediated mTOR signaling pathway also played an important role in the proliferation of breast cancer cells. STING or TBK1 inhibition markedly attenuated the MARCH1 deficiency induced by faster proliferation of breast cancer cells. This may explain the positive role of STING/TBK1 in tumor progression of breast cancer.

The cell experiments in our study also showed that MARCH1 mediated the TBK1-mTOR signaling pathway to both respond to insulin and EGF stimulation in breast cancer cells. This finding is different from that of a previous study by Bodur et al. [22] in mouse embryonic fibroblasts that demonstrated TBK1 mediated the mTOR signaling pathway only in response to EGF stimulation. In contrast, Tooley et al. [23] confirmed that both insulin and EGF stimulated TBK1 to mediate the mTOR signaling pathway in mouse embryonic fibroblasts. We consider this discrepancy may be due to the different experimental conditions used in the two studies.

## Conclusions

In summary, we demonstrated that MARCH1 suppresses the TBK1-mTOR signaling pathway by promoting ubiquitination of TBK1. MARCH1 interacts with TBK1 and promotes K63-linked ubiquitination, which subsequently decreases the interaction with mTOR, resulting in suppression of the mTOR signaling pathway. Importantly, a deficiency of MARCH1 promotes the proliferation and migration of breast cancer cells. This study therefore sheds light on the mechanism of the MARCH1-STING-TBK1-mTOR signaling pathway and offers a theoretical basis for the treatment of breast cancer.

### Supplementary Information

Supplementary Material 1: Supplementary Figure 1. (A-E) Quantitation of p-S6K1 T389 and p-AKT1 S473 in Fig. 1A-E. (F) Quantitation of p-ULK1S757 and p-SGK1 S422 in Fig. 1F. (G-N) Quantitation of p-S6K1 T389 and p-AKT1 S473 in Fig. 1G-N. The graph quantitates three independent experiments each with *n* = 1 (*n* = 3 total). The data are expressed as mean (SD). *P ≤ 0.05, **P ≤ 0.01. Supplementary Figure 2. (A-C) Quantitation of p-TBK1 S172 in Fig. 2A-C. (D-F) Quantitation of p-S6K1 T389 and p-TBK1 S172 in Fig. 2D-F. (G-I) Quantitation of p-AKT1 S473 and p-TBK1 S172 in Fig. 2G-I. (J-L) Quantitation of p-AKT1 S473, p-S6K1 T389, and p-TBK1 S172 in Fig. 2J-L. (N) Quantitation of p-S6K1 T389 and p-AKT1 S473 in Fig. 2N. (O) Quantitation of p-ULK1S757, p-SGK1 S422, and p-TBK1 S172 in Fig. 1F. The chart quantitates three independent experiments each with *n* = 1 (*n* = 3 total). The data are expressed as mean (SD). *P ≤ 0.05, **P ≤ 0.01. Supplementary Figure 3. (A-D) Quantitation of p-S6K1 T389 and p-TBK1 S172 in Fig. 3A-D. (E-H) Quantitation of p-AKT1 S473 and p-TBK1 S172 in Fig. 3E-H. (I-J) Quantitation of p-AKT1 S473, p-S6K1 T389, and p-TBK1 S172 in Fig. 3I-J. (K) Quantitation of p-S6K1 T389 and p-AKT1 S473 in Fig. 3K. The chart quantitates three independent experiments each with *n* = 1 (*n* = 3 total). The data are expressed as mean (SD). *P ≤ 0.05, **P ≤ 0.01. Supplementary Figure 4. (A-B) Quantitation of STING-myc in Fig. 4B-C. (C-E) Quantitation of mTOR in Fig. 4D-F. (F) Quantitation of S6K1 in Fig. 4G. (G) Quantitation of mTOR and S6K1 in Fig. 4H. (H) Quantitation of mTOR in Fig. 4I. (I) Quantitation of STING-myc in Fig. 5A. (J) Quantitation of mTOR in Fig. 5J. (K) Quantitation of p-AKT1 S473, p-S6K1 T389, and p-TBK1 S172 in Fig. 5K. The graph quantitates three independent experiments each with *n* = 1 (*n* = 3 total). The data are expressed as mean (SD). *P ≤ 0.05, **P ≤ 0.01.

## Data Availability

No datasets were generated or analysed during the current study.

## Abbreviations

mTORC1: Mammalian target of rapamycin complex1
TBK1: TANK-binding kinase 1
RAPTOR: Regulatory associated protein of mTOR
PRAS40: Proline-rich AKT substrate 40 kDa
PI3K: Phosphatidylinositol 3-kinase
cGAS: CGAMP synthase
STING: Stimulator of IFN genes
cGAMP: Cyclic-GMP-AMP
ERGIC: ER-Golgi intermediate compartment
IRF3: Interferon regulatory factor 3
NSCLC: Non-small cell lung cancer
MARCH1: Membrane-associated RING-CH-1
DCs: Dendritic cells
MHC-II: Major histocompatibility complex class II
HCMV: Human cytomegalovirus

## References

[1] Saxton RA, Sabatini DM. mTOR signaling in growth, metabolism, and disease. Cell. 2017;169(2):361–71. 28388417 10.1016/j.cell.2017.03.035

[2] Vander Haar E, Lee SI, Bandhakavi S, Griffin TJ, Kim DH. Insulin signalling to mTOR mediated by the Akt/PKB substrate PRAS40. Nat Cell Biol. 2007;9(3):316–23. 17277771 10.1038/ncb1547

[3] Hara K, Maruki Y, Long X, Yoshino K, Oshiro N, Hidayat S, Tokunaga C, Avruch J, Yonezawa K. Raptor, a binding partner of target of rapamycin (TOR), mediates TOR action. Cell. 2002;110(2):177–89. 12150926 10.1016/S0092-8674(02)00833-4

[4] Loewith R, Jacinto E, Wullschleger S, Lorberg A, Crespo JL, Bonenfant D, Oppliger W, Jenoe P, Hall MN. Two TOR complexes, only one of which is rapamycin sensitive, have distinct roles in cell growth control. Mol Cell. 2002;10(3):457–68. 12408816 10.1016/S1097-2765(02)00636-6

[5] Sancak Y, Thoreen CC, Peterson TR, Lindquist RA, Kang SA, Spooner E, Carr SA, Sabatini DM. PRAS40 is an insulin-regulated inhibitor of the mTORC1 protein kinase. Mol Cell. 2007;25(6):903–15. 17386266 10.1016/j.molcel.2007.03.003

[6] Jacinto E, Loewith R, Schmidt A, Lin S, Ruegg MA, Hall A, Hall MN. Mammalian TOR complex 2 controls the actin cytoskeleton and is rapamycin insensitive. Nat Cell Biol. 2004;6(11):1122–8. 15467718 10.1038/ncb1183

[7] Sarbassov DD, Ali SM, Kim DH, Guertin DA, Latek RR, Erdjument-Bromage H, Tempst P, Sabatini DM. Rictor, a novel binding partner of mTOR, defines a rapamycin-insensitive and raptor-independent pathway that regulates the cytoskeleton. Curr Biol. 2004;14(14):1296–302. 15268862 10.1016/j.cub.2004.06.054

[8] Frias MA, Thoreen CC, Jaffe JD, Schroder W, Sculley T, Carr SA, Sabatini DM. mSin1 is necessary for Akt/PKB phosphorylation, and its isoforms define three distinct mTORC2s. Curr Biol. 2006;16(18):1865–70. 16919458 10.1016/j.cub.2006.08.001

[9] Jacinto E, Facchinetti V, Liu D, Soto N, Wei S, Jung SY, Huang Q, Qin J, Su B. SIN1/MIP1 maintains rictor-mTOR complex integrity and regulates Akt phosphorylation and substrate specificity. Cell. 2006;127(1):125–37. 16962653 10.1016/j.cell.2006.08.033

[10] Isotani S, Hara K, Tokunaga C, Inoue H, Avruch J, Yonezawa K. Immunopurified mammalian target of rapamycin phosphorylates and activates p70 S6 kinase alpha in vitro. J Biol Chem. 1999;274(48):34493–8. 10567431 10.1074/jbc.274.48.34493

[11] Nazio F, Strappazzon F, Antonioli M, Bielli P, Cianfanelli V, Bordi M, Gretzmeier C, Dengjel J, Piacentini M, Fimia GM, et al. mTOR inhibits autophagy by controlling ULK1 ubiquitylation, self-association and function through AMBRA1 and TRAF6. Nat Cell Biol. 2013;15(4):406–16. 23524951 10.1038/ncb2708

[12] Zinzalla V, Stracka D, Oppliger W, Hall MN. Activation of mTORC2 by association with the ribosome. Cell. 2011;144(5):757–68. 21376236 10.1016/j.cell.2011.02.014

[13] Ikenoue T, Inoki K, Yang Q, Zhou X, Guan KL. Essential function of TORC2 in PKC and Akt turn motif phosphorylation, maturation and signalling. EMBO J. 2008;27(14):1919–31. 18566587 10.1038/emboj.2008.119 PMC2486275

[14] Sarbassov DD, Guertin DA, Ali SM, Sabatini DM. Phosphorylation and regulation of Akt/PKB by the rictor-mTOR complex. Science. 2005;307(5712):1098–101. 15718470 10.1126/science.1106148

[15] García-Martínez JM, Alessi DR. mTOR complex 2 (mTORC2) controls hydrophobic motif phosphorylation and activation of serum- and glucocorticoid-induced protein kinase 1 (SGK1). Biochem J. 2008;416(3):375–85. 18925875 10.1042/BJ20081668

[16] Prabakaran T, Bodda C, Krapp C, Zhang BC, Christensen MH, Sun C, Reinert L, Cai Y, Jensen SB, Skouboe MK, et al. Attenuation of cGAS-STING signaling is mediated by a p62/SQSTM1-dependent autophagy pathway activated by TBK1. EMBO J. 2018;37(8):e97858. 29496741 10.15252/embj.201797858 PMC5897779

[17] Hasan M, Gonugunta VK, Dobbs N, Ali A, Palchik G, Calvaruso MA, DeBerardinis RJ, Yan N. Chronic innate immune activation of TBK1 suppresses mTORC1 activity and dysregulates cellular metabolism. Proc Natl Acad Sci U S A. 2017;114(4):746–51. 28069950 10.1073/pnas.1611113114 PMC5278463

[18] Runde AP, Mack R, S JP, Zhang J. The role of TBK1 in cancer pathogenesis and anticancer immunity. J Exp Clin Cancer Res. 2022;41(1):135. 35395857 10.1186/s13046-022-02352-y PMC8994244

[19] Galluzzi L, Vanpouille-Box C, Bakhoum SF, Demaria S. SnapShot: CGAS-STING signaling. Cell. 2018;173(1):276-276.e271. 29570996 10.1016/j.cell.2018.03.015

[20] Diner EJ, Burdette DL, Wilson SC, Monroe KM, Kellenberger CA, Hyodo M, Hayakawa Y, Hammond MC, Vance RE. The innate immune DNA sensor cGAS produces a noncanonical cyclic dinucleotide that activates human STING. Cell Rep. 2013;3(5):1355–61. 23707065 10.1016/j.celrep.2013.05.009 PMC3706192

[21] Wang C, Wang X, Veleeparambil M, Kessler PM, Willard B, Chattopadhyay S, Sen GC. EGFR-mediated tyrosine phosphorylation of STING determines its trafficking route and cellular innate immunity functions. EMBO J. 2020;39(22):e104106. 32926474 10.15252/embj.2019104106 PMC7667877

[22] Bodur C, Kazyken D, Huang K, EkimUstunel B, Siroky KA, Tooley AS, Gonzalez IE, Foley DH, Acosta-Jaquez HA, Barnes TM, et al. The IKK-related kinase TBK1 activates mTORC1 directly in response to growth factors and innate immune agonists. EMBO J. 2018;37(1):19–38. 29150432 10.15252/embj.201696164 PMC5753041

[23] Tooley AS, Kazyken D, Bodur C, Gonzalez IE, Fingar DC. The innate immune kinase TBK1 directly increases mTORC2 activity and downstream signaling to Akt. J Biol Chem. 2021;297(2):100942. 34245780 10.1016/j.jbc.2021.100942 PMC8342794

[24] Cooper JM, Ou YH, McMillan EA, Vaden RM, Zaman A, Bodemann BO, Makkar G, Posner BA, White MA. TBK1 provides context-selective support of the activated AKT/mTOR pathway in lung cancer. Cancer Res. 2017;77(18):5077–94. 28716898 10.1158/0008-5472.CAN-17-0829 PMC5833933

[25] Lin H, Li S, Shu HB. The membrane-associated MARCH E3 ligase family: emerging roles in immune regulation. Front Immunol. 2019;10:1751. 31404274 10.3389/fimmu.2019.01751 PMC6669941

[26] Matsuki Y, Ohmura-Hoshino M, Goto E, Aoki M, Mito-Yoshida M, Uematsu M, Hasegawa T, Koseki H, Ohara O, Nakayama M, et al. Novel regulation of MHC class II function in B cells. EMBO J. 2007;26(3):846–54. 17255932 10.1038/sj.emboj.7601556 PMC1794403

[27] Martin M, Sandhu P, Kumar R, Buchkovich NJ. The immune-specific E3 ubiquitin ligase MARCH1 is upregulated during human cytomegalovirus infection to regulate iron levels. J Virol. 2022;96(6):e0180621. 35045264 10.1128/jvi.01806-21 PMC8941913

[28] Wu J, Xia L, Yao X, Yu X, Tumas KC, Sun W, Cheng Y, He X, Peng YC, Singh BK, et al. The E3 ubiquitin ligase MARCH1 regulates antimalaria immunity through interferon signaling and T cell activation. Proc Natl Acad Sci U S A. 2020;117(28):16567–78. 32606244 10.1073/pnas.2004332117 PMC7368286

[29] Thoreen CC, Kang SA, Chang JW, Liu Q, Zhang J, Gao Y, Reichling LJ, Sim T, Sabatini DM, Gray NS. An ATP-competitive mammalian target of rapamycin inhibitor reveals rapamycin-resistant functions of mTORC1. J Biol Chem. 2009;284(12):8023–32. 19150980 10.1074/jbc.M900301200 PMC2658096

[30] Kong D, Yamori T. ZSTK474 is an ATP-competitive inhibitor of class I phosphatidylinositol 3 kinase isoforms. Cancer Sci. 2007;98(10):1638–42. 17711503 10.1111/j.1349-7006.2007.00580.x PMC11158993

[31] Thomson DW, Poeckel D, Zinn N, Rau C, Strohmer K, Wagner AJ, Graves AP, Perrin J, Bantscheff M, Duempelfeld B, et al. Discovery of GSK8612, a highly selective and potent TBK1 inhibitor. ACS Med Chem Lett. 2019;10(5):780–5. 31097999 10.1021/acsmedchemlett.9b00027 PMC6512007

[32] Zhao BY, Du FL, Xu PB, Shu C, Sankaran B, Bell SL, Liu MM, Lei YJ, Gao XS, Fu XF, et al. A conserved PLPLRT/SD motif of STING mediates the recruitment and activation of TBK1. Nature. 2019;569(7758):718-#x0002B; 31118511 10.1038/s41586-019-1228-x PMC6596994

[33] Reus JB, Trivino-Soto GS, Wu LI, Kokott K, Lim ES. SV40 large T antigen is not responsible for the loss of STING in 293T cells but can inhibit cGAS-STING interferon induction. Viruses. 2020;12(2):137. 31991682 10.3390/v12020137 PMC7077178

[34] Sun L, Wu J, Du F, Chen X, Chen ZJ. Cyclic GMP-AMP synthase is a cytosolic DNA sensor that activates the type I interferon pathway. Science. 2013;339(6121):786–91. 23258413 10.1126/science.1232458 PMC3863629

[35] Linares JF, Duran A, Yajima T, Pasparakis M, Moscat J, Diaz-Meco MT. K63 polyubiquitination and activation of mTOR by the p62-TRAF6 complex in nutrient-activated cells. Mol Cell. 2013;51(3):283–96. 23911927 10.1016/j.molcel.2013.06.020 PMC3971544

[36] Baravalle G, Park H, McSweeney M, Ohmura-Hoshino M, Matsuki Y, Ishido S, Shin JS. Ubiquitination of CD86 is a key mechanism in regulating antigen presentation by dendritic cells. J Immunol. 2011;187(6):2966–73. 21849678 10.4049/jimmunol.1101643 PMC4496154

[37] Su Y, Feng W, Zhong G, Ya Y, Du Z, Shi J, Chen L, Dong W, Lin T. ciRs-6 upregulates March1 to suppress bladder cancer growth by sponging miR-653. Aging (Albany NY). 2019;11(23):11202–23. 31819015 10.18632/aging.102525 PMC6932879

[38] Xu Z, Liu J, Liu Z, Zhang H. MARCH1 as a novel immune-related prognostic biomarker that shapes an inflamed tumor microenvironment in lung adenocarcinoma. Front Oncol. 2022;12:1008753. 36313698 10.3389/fonc.2022.1008753 PMC9606618

[39] Yu J, Zhou X, Chang M, Nakaya M, Chang JH, Xiao Y, Lindsey JW, Dorta-Estremera S, Cao W, Zal A, et al. Regulation of T-cell activation and migration by the kinase TBK1 during neuroinflammation. Nat Commun. 2015;6:6074. 25606824 10.1038/ncomms7074 PMC4302769

[40] Pilli M, Arko-Mensah J, Ponpuak M, Roberts E, Master S, Mandell MA, Dupont N, Ornatowski W, Jiang S, Bradfute SB, et al. TBK-1 promotes autophagy-mediated antimicrobial defense by controlling autophagosome maturation. Immunity. 2012;37(2):223–34. 22921120 10.1016/j.immuni.2012.04.015 PMC3428731

[41] Thurston TL, Boyle KB, Allen M, Ravenhill BJ, Karpiyevich M, Bloor S, Kaul A, Noad J, Foeglein A, Matthews SA, et al. Recruitment of TBK1 to cytosol-invading Salmonella induces WIPI2-dependent antibacterial autophagy. EMBO J. 2016;35(16):1779–92. 27370208 10.15252/embj.201694491 PMC5010046

[42] Song G, Liu B, Li Z, Wu H, Wang P, Zhao K, Jiang G, Zhang L, Gao C. E3 ubiquitin ligase RNF128 promotes innate antiviral immunity through K63-linked ubiquitination of TBK1. Nat Immunol. 2016;17(12):1342–51. 27776110 10.1038/ni.3588

[43] Fang R, Jiang QF, Zhou X, Wang CG, Guan YK, Tao JL, Xi JZ, Feng JM, Jiang ZF. MAVS activates TBK1 and IKKε through TRAFs in NEMO dependent and independent manner. PLoS Pathog. 2017;13(11):e1006720. 29125880 10.1371/journal.ppat.1006720 PMC5699845

[44] Wang C, Chen T, Zhang J, Yang M, Li N, Xu X, Cao X. The E3 ubiquitin ligase Nrdp1 ‘preferentially’ promotes TLR-mediated production of type I interferon. Nat Immunol. 2009;10(7):744–52. 19483718 10.1038/ni.1742

[45] Cui J, Li Y, Zhu L, Liu D, Songyang Z, Wang HY, Wang RF. NLRP4 negatively regulates type I interferon signaling by targeting the kinase TBK1 for degradation via the ubiquitin ligase DTX4. Nat Immunol. 2012;13(4):387–95. 22388039 10.1038/ni.2239 PMC3760161

[46] Zhao P, Wong KI, Sun XL, Reilly SM, Uhm M, Liao ZJ, Skorobogatko Y, Saltiel AR. TBK1 at the crossroads of inflammation and energy homeostasis in adipose tissue. Cell. 2018;172(4):731-#x0002B; 29425491 10.1016/j.cell.2018.01.007 PMC5808582

[47] Clark K, Peggie M, Plater L, Sorcek RJ, Young ERR, Madwed JB, Hough J, McIver EG, Cohen P. Novel cross-talk within the IKK family controls innate immunity. Biochem J. 2011;434:93–104. 21138416 10.1042/BJ20101701

[48] Wang L, Li S, Dorf ME. NEMO binds ubiquitinated TANK-binding kinase 1 (TBK1) to regulate innate immune responses to RNA viruses. PLoS One. 2012;7(9):e43756. 23028469 10.1371/journal.pone.0043756 PMC3445589

[49] Tu D, Zhu Z, Zhou AY, Yun CH, Lee KE, Toms AV, Li Y, Dunn GP, Chan E, Thai T, et al. Structure and ubiquitination-dependent activation of TANK-binding kinase 1. Cell Rep. 2013;3(3):747–58. 23453972 10.1016/j.celrep.2013.01.033 PMC3863638

[50] Lin M, Zhao Z, Yang Z, Meng Q, Tan P, Xie W, Qin Y, Wang RF, Cui J. USP38 inhibits type I interferon signaling by editing TBK1 ubiquitination through NLRP4 signalosome. Mol Cell. 2016;64(2):267–81. 27692986 10.1016/j.molcel.2016.08.029

[51] Xie X, Zhang D, Zhao B, Lu MK, You M, Condorelli G, Wang CY, Guan KL. IkappaB kinase epsilon and TANK-binding kinase 1 activate AKT by direct phosphorylation. Proc Natl Acad Sci U S A. 2011;108(16):6474–9. 21464307 10.1073/pnas.1016132108 PMC3081021

[52] Ou YH, Torres M, Ram R, Formstecher E, Roland C, Cheng T, Brekken R, Wurz R, Tasker A, Polverino T, et al. TBK1 directly engages Akt/PKB survival signaling to support oncogenic transformation. Mol Cell. 2011;41(4):458–70. 21329883 10.1016/j.molcel.2011.01.019 PMC3073833

[53] Kim JK, Jung Y, Wang J, Joseph J, Mishra A, Hill EE, Krebsbach PH, Pienta KJ, Shiozawa Y, Taichman RS. TBK1 regulates prostate cancer dormancy through mTOR inhibition. Neoplasia. 2013;15(9):1064–74. 24027431 10.1593/neo.13402 PMC3769885

[54] Antonia RJ, Castillo J, Herring LE, Serafin DS, Liu P, Graves LM, Baldwin AS, Hagan RS. TBK1 limits mTORC1 by promoting phosphorylation of raptor Ser877. Sci Rep. 2019;9(1):13470. 31530866 10.1038/s41598-019-49707-8 PMC6748941

[55] Ye M, Hu Y, Zhao B, Mou Q, Ni Y, Luo J, Li L, Zhang H, Zhao Y. TBK1 knockdown alleviates axonal transport deficits in retinal ganglion cells via mTORC1 activation in a retinal damage mouse model. Invest Ophthalmol Vis Sci. 2023;64(10):1. 37395713 10.1167/iovs.64.10.1 PMC10324417

[56] Wang H, Hu S, Chen X, Shi H, Chen C, Sun L, Chen ZJ. cGAS is essential for the antitumor effect of immune checkpoint blockade. Proc Natl Acad Sci U S A. 2017;114(7):1637–42. 28137885 10.1073/pnas.1621363114 PMC5320994

[57] Corrales L, Glickman LH, McWhirter SM, Kanne DB, Sivick KE, Katibah GE, Woo SR, Lemmens E, Banda T, Leong JJ, et al. Direct activation of STING in the tumor microenvironment leads to potent and systemic tumor regression and immunity. Cell Rep. 2015;11(7):1018–30. 25959818 10.1016/j.celrep.2015.04.031 PMC4440852

[58] Demaria O, De Gassart A, Coso S, Gestermann N, Di Domizio J, Flatz L, Gaide O, Michielin O, Hwu P, Petrova TV, et al. STING activation of tumor endothelial cells initiates spontaneous and therapeutic antitumor immunity. Proc Natl Acad Sci U S A. 2015;112(50):15408–13. 26607445 10.1073/pnas.1512832112 PMC4687570

[59] Deng L, Liang H, Xu M, Yang X, Burnette B, Arina A, Li XD, Mauceri H, Beckett M, Darga T, et al. STING-dependent cytosolic DNA sensing promotes radiation-induced type I interferon-dependent antitumor immunity in immunogenic tumors. Immunity. 2014;41(5):843–52. 25517616 10.1016/j.immuni.2014.10.019 PMC5155593

[60] Woo SR, Fuertes MB, Corrales L, Spranger S, Furdyna MJ, Leung MY, Duggan R, Wang Y, Barber GN, Fitzgerald KA, et al. STING-dependent cytosolic DNA sensing mediates innate immune recognition of immunogenic tumors. Immunity. 2014;41(5):830–42. 25517615 10.1016/j.immuni.2014.10.017 PMC4384884

[61] An X, Zhu Y, Zheng T, Wang G, Zhang M, Li J, Ji H, Li S, Yang S, Xu D, et al. An analysis of the expression and association with immune cell infiltration of the cGAS/STING pathway in pan-cancer. Mol Ther Nucleic Acids. 2019;14:80–9. 30583098 10.1016/j.omtn.2018.11.003 PMC6305687

[62] Jiang Y, Chen S, Li Q, Liang J, Lin W, Li J, Liu Z, Wen M, Cao M, Hong J. TANK-binding kinase 1 (TBK1) serves as a potential target for hepatocellular carcinoma by enhancing tumor immune infiltration. Front Immunol. 2021;12:612139. 33679751 10.3389/fimmu.2021.612139 PMC7930497

[63] Xiao Y, Zou Q, Xie X, Liu T, Li HS, Jie Z, Jin J, Hu H, Manyam G, Zhang L, et al. The kinase TBK1 functions in dendritic cells to regulate T cell homeostasis, autoimmunity, and antitumor immunity. J Exp Med. 2017;214(5):1493–507. 28356390 10.1084/jem.20161524 PMC5413337

[64] Tang CH, Zundell JA, Ranatunga S, Lin C, Nefedova Y, Del Valle JR, Hu CC. Agonist-mediated activation of STING induces apoptosis in malignant B cells. Cancer Res. 2016;76(8):2137–52. 26951929 10.1158/0008-5472.CAN-15-1885 PMC4873432

[65] Ka NL, Lim GY, Hwang S, Kim SS, Lee MO. IFI16 inhibits DNA repair that potentiates type-I interferon-induced antitumor effects in triple negative breast cancer. Cell Rep. 2021;37(12):110138. 34936865 10.1016/j.celrep.2021.110138

[66] Cheradame L, Guerrera IC, Gaston J, Schmitt A, Jung V, Pouillard M, Radosevic-Robin N, Modesti M, Judde J-G, Cairo S, et al. STING promotes breast cancer cell survival by an inflammatory-independent nuclear pathway enhancing the DNA damage response. bioRxiv. 2020.2007.2011.196790.

[67] Vasiyani H, Mane M, Rana K, Shinde A, Roy M, Singh J, Gohel D, Currim F, Srivastava R, Singh R. DNA damage induces STING mediated IL-6-STAT3 survival pathway in triple-negative breast cancer cells and decreased survival of breast cancer patients. Apoptosis. 2022;27(11–12):961–78. 36018392 10.1007/s10495-022-01763-8

[68] Cheradame L, Guerrera IC, Gaston J, Schmitt A, Jung V, Goudin N, Pouillard M, Radosevic-Robin N, Modesti M, Judde JG, et al. STING protects breast cancer cells from intrinsic and genotoxic-induced DNA instability via a non-canonical, cell-autonomous pathway. Oncogene. 2021;40(49):6627–40. 34625708 10.1038/s41388-021-02037-4

[69] Chen Q, Boire A, Jin X, Valiente M, Er EE, Lopez-Soto A, Jacob L, Patwa R, Shah H, Xu K, et al. Carcinoma-astrocyte gap junctions promote brain metastasis by cGAMP transfer. Nature. 2016;533(7604):493–8. 27225120 10.1038/nature18268 PMC5021195

[70] Gaston J, Cheradame L, Yvonnet V, Deas O, Poupon MF, Judde JG, Cairo S, Goffin V. Intracellular STING inactivation sensitizes breast cancer cells to genotoxic agents. Oncotarget. 2016;7(47):77205–24. 27791205 10.18632/oncotarget.12858 PMC5363581

[71] Shen RR, Hahn WC. Emerging roles for the non-canonical IKKs in cancer. Oncogene. 2011;30(6):631–41. 21042276 10.1038/onc.2010.493 PMC3235643

[72] Jiang Q, Guan Y, Zheng J, Lu H. TBK1 promotes thyroid cancer progress by activating the PI3K/Akt/mTOR signaling pathway. Immun Inflamm Dis. 2023;11(3):e796. 36988258 10.1002/iid3.796 PMC10013413

[73] Barbie DA, Tamayo P, Boehm JS, Kim SY, Moody SE, Dunn IF, Schinzel AC, Sandy P, Meylan E, Scholl C, et al. Systematic RNA interference reveals that oncogenic KRAS-driven cancers require TBK1. Nature. 2009;462(7269):108–12. 19847166 10.1038/nature08460 PMC2783335

[74] Wei C, Cao Y, Yang X, Zheng Z, Guan K, Wang Q, Tai Y, Zhang Y, Ma S, Cao Y, et al. Elevated expression of TANK-binding kinase 1 enhances tamoxifen resistance in breast cancer. Proc Natl Acad Sci U S A. 2014;111(5):E601-610. 24449872 10.1073/pnas.1316255111 PMC3918824

