# Supplementary material for: MARCH1 negatively regulates TBK1-mTOR signaling pathway by ubiquitinating TBK1
Source: BMC Cancer. 2024 Jul 26;24:902. doi: 10.1186/s12885-024-12667-y (PMC11282859; doi:10.1186/s12885-024-12667-y)
Supplement: Supplementary file 1 — Supplementary Material 1: Supplementary Figure 1. (A-E) Quantitation of p-S6K1 T389 and p-AKT1 S473 in Fig. 1A-E. (F) Quantitation of p-ULK1S757 and p-SGK1 S422 in Fig. 1F. (G-N) Quantitation of p-S6K1 T389 and p-AKT1 S473 in Fig. 1G-N. The graph quantitates three independent experiments each with n = 1 (n = 3 total). The data are expressed as mean (SD). *P ≤ 0.05, **P ≤ 0.01. Supplementary Figure 2. (A-C) Quantitation of p-TBK1 S172 in Fig. 2A-C. (D-F) Quantitation of p-S6K1 T389 and p-TBK1 S172 in Fig. 2D-F. (G-I) Quantitation of p-AKT1 S473 and p-TBK1 S172 in Fig. 2G-I. (J-L) Quantitation of p-AKT1 S473, p-S6K1 T389, and p-TBK1 S172 in Fig. 2J-L. (N) Quantitation of p-S6K1 T389 and p-AKT1 S473 in Fig. 2N. (O) Quantitation of p-ULK1S757, p-SGK1 S422, and p-TBK1 S172 in Fig. 1F. The chart quantitates three independent experiments each with n = 1 (n = 3 total). The data are expressed as mean (SD). *P ≤ 0.05, **P ≤ 0.01. Supplementary Figure 3. (A-D) Quantitation of p-S6K1 T389 and p-TBK1 S172 in Fig. 3A-D. (E-H) Quantitation of p-AKT1 S473 and p-TBK1 S172 in Fig. 3E-H. (I-J) Quantitation of p-AKT1 S473, p-S6K1 T389, and p-TBK1 S172 in Fig. 3I-J. (K) Quantitation of p-S6K1 T389 and p-AKT1 S473 in Fig. 3K. The chart quantitates three independent experiments each with n = 1 (n = 3 total). The data are expressed as mean (SD). *P ≤ 0.05, **P ≤ 0.01. Supplementary Figure 4. (A-B) Quantitation of STING-myc in Fig. 4B-C. (C-E) Quantitation of mTOR in Fig. 4D-F. (F) Quantitation of S6K1 in Fig. 4G. (G) Quantitation of mTOR and S6K1 in Fig. 4H. (H) Quantitation of mTOR in Fig. 4I. (I) Quantitation of STING-myc in Fig. 5A. (J) Quantitation of mTOR in Fig. 5J. (K) Quantitation of p-AKT1 S473, p-S6K1 T389, and p-TBK1 S172 in Fig. 5K. The graph quantitates three independent experiments each with n = 1 (n = 3 total). The data are expressed as mean (SD). *P ≤ 0.05, **P ≤ 0.01. [file 12885_2024_12667_MOESM1_ESM.zip › Supplementary Information/supplemental figures.pptx]

## Slide 1
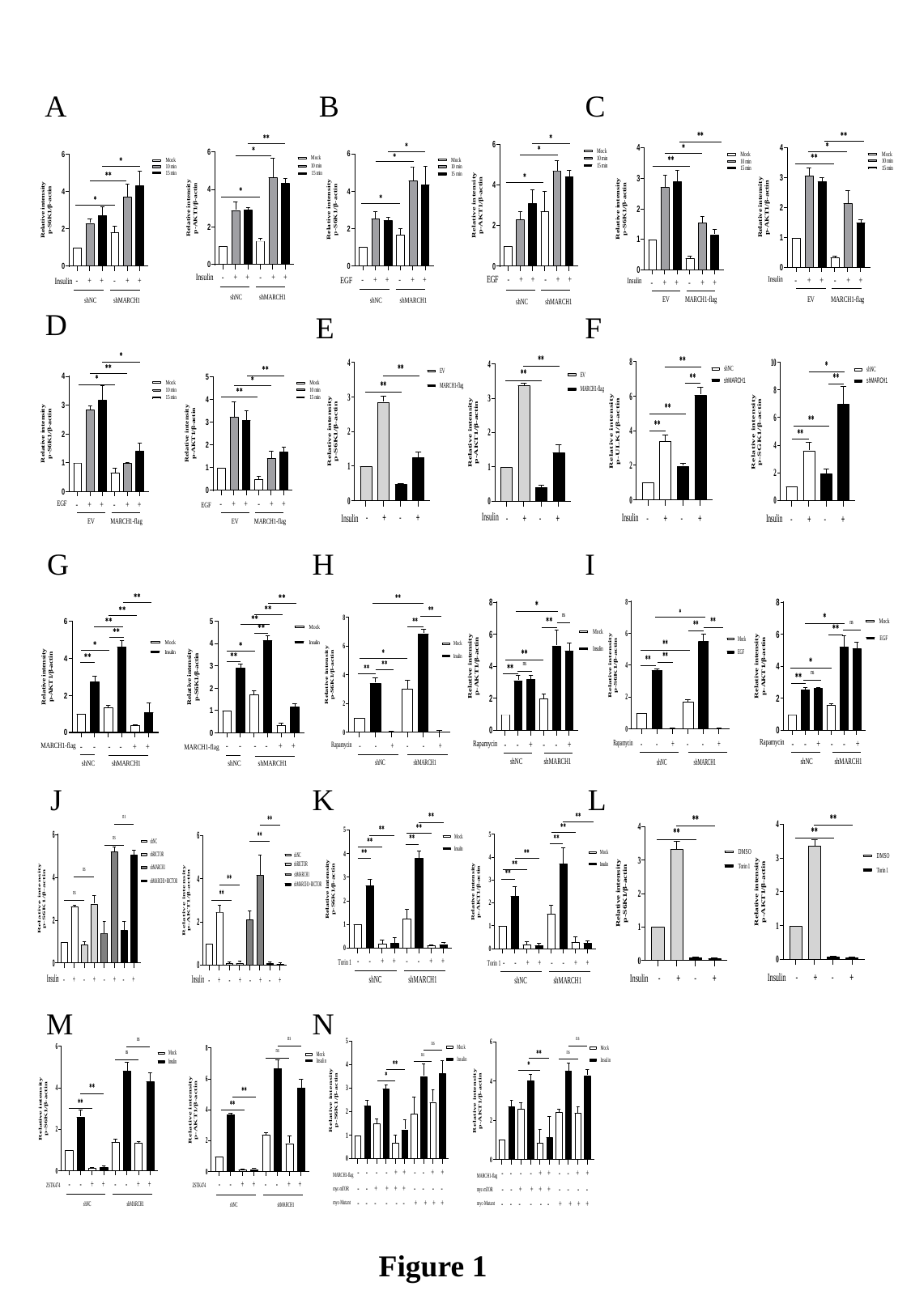

A
B
C
D
F
E
G
H
I
J
K
L
M
N
Figure 1

## Slide 2
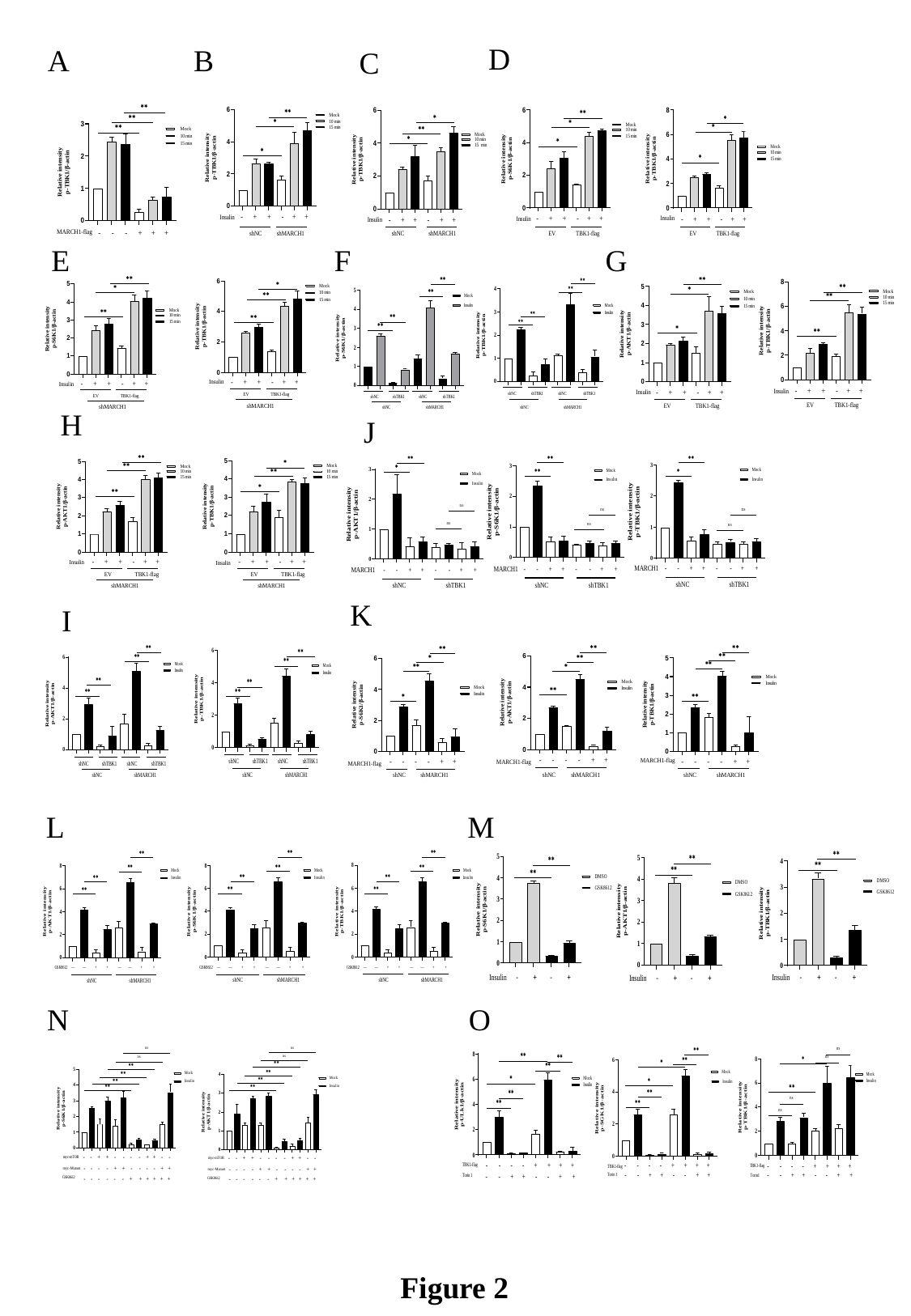

D
A
B
C
E
F
G
H
J
K
I
L
M
N
O
Figure 2

## Slide 3
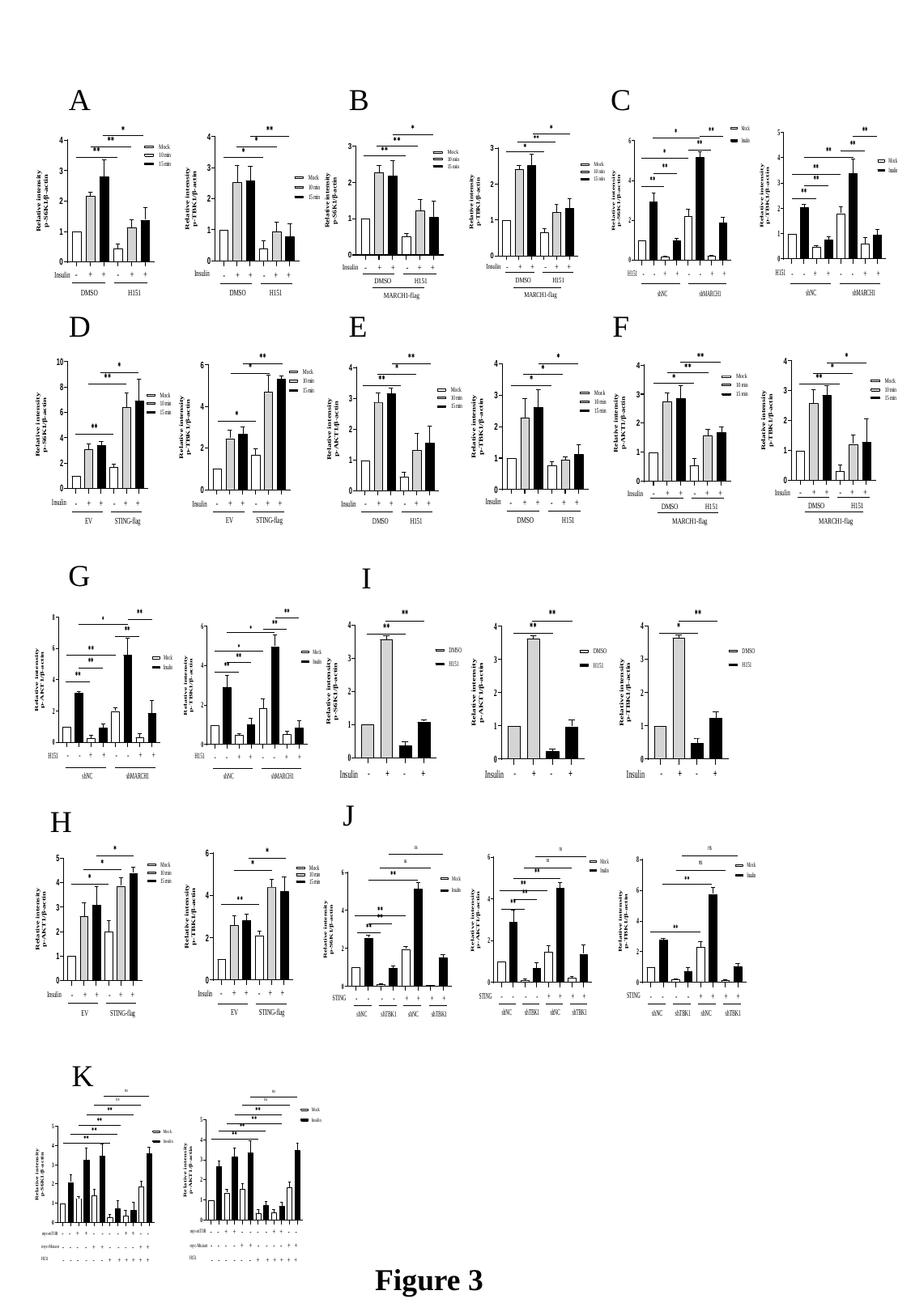

A
B
C
D
E
F
G
I
J
H
K
Figure 3

## Slide 4
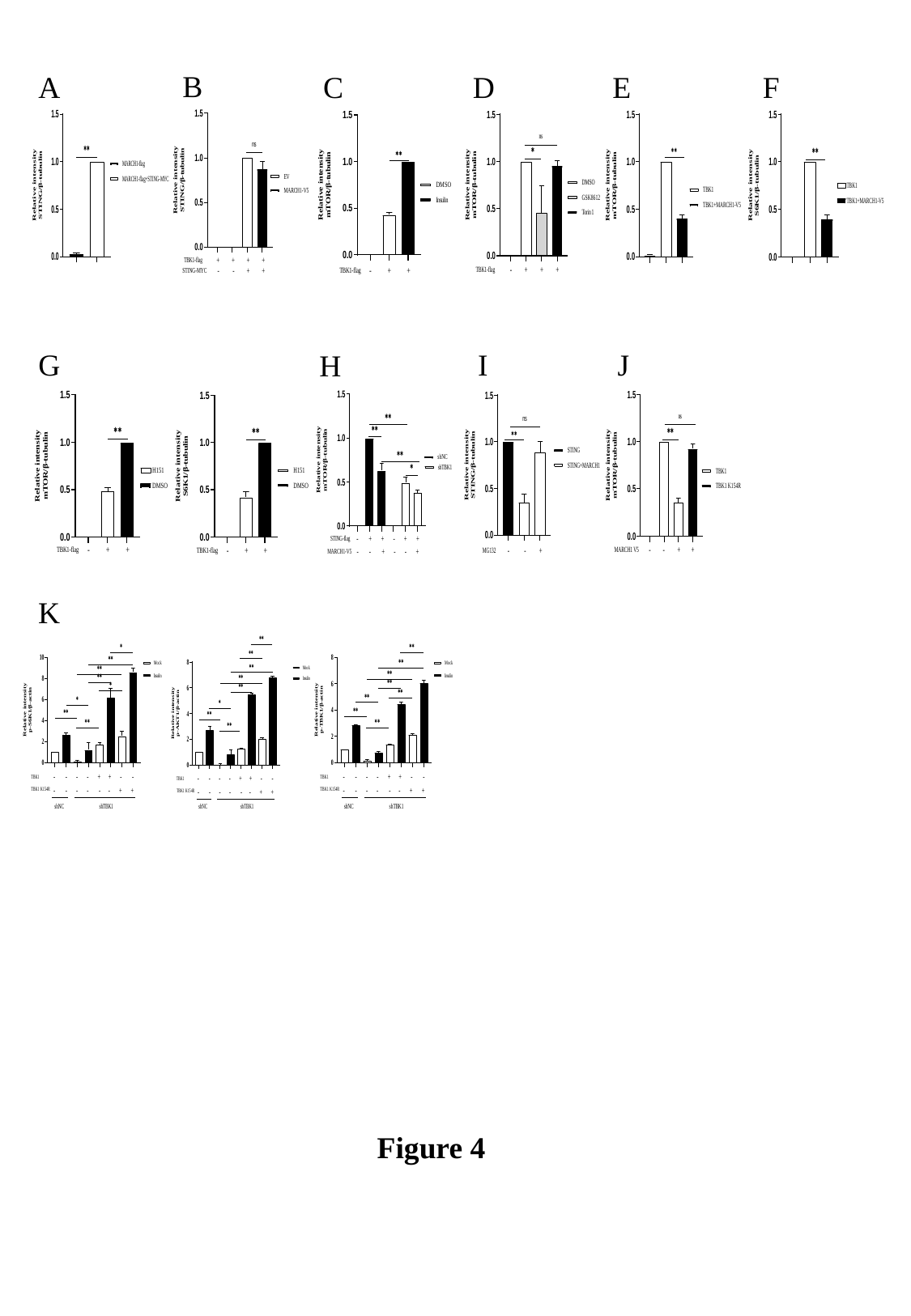

B
E
F
A
C
D
G
I
J
H
K
Figure 4
